# Environmental uncertainty and the advantage of impulsive choice strategies

**DOI:** 10.1371/journal.pcbi.1010873

**Published:** 2023-01-30

**Authors:** Diana C. Burk, Bruno B. Averbeck

**Affiliations:** Laboratory of Neuropsychology, National Institute of Mental Health, National Institutes of Health, Bethesda, Maryland, United States of America; Dartmouth College, UNITED STATES

## Abstract

Choice impulsivity is characterized by the choice of immediate, smaller reward options over future, larger reward options, and is often thought to be associated with negative life outcomes. However, some environments make future rewards more uncertain, and in these environments impulsive choices can be beneficial. Here we examined the conditions under which impulsive vs. non-impulsive decision strategies would be advantageous. We used Markov Decision Processes (MDPs) to model three common decision-making tasks: Temporal Discounting, Information Sampling, and an Explore-Exploit task. We manipulated environmental variables to create circumstances where future outcomes were relatively uncertain. We then manipulated the discount factor of an MDP agent, which affects the value of immediate versus future rewards, to model impulsive and non-impulsive behavior. This allowed us to examine the performance of impulsive and non-impulsive agents in more or less predictable environments. In Temporal Discounting, we manipulated the transition probability to delayed rewards and found that the agent with the lower discount factor (i.e. the impulsive agent) collected more average reward than the agent with a higher discount factor (the non-impulsive agent) by selecting immediate reward options when the probability of receiving the future reward was low. In the Information Sampling task, we manipulated the amount of information obtained with each sample. When sampling led to small information gains, the impulsive MDP agent collected more average reward than the non-impulsive agent. Third, in the Explore-Exploit task, we manipulated the substitution rate for novel options. When the substitution rate was high, the impulsive agent again performed better than the non-impulsive agent, as it explored the novel options less and instead exploited options with known reward values. The results of these analyses show that impulsivity can be advantageous in environments that are unexpectedly uncertain.

## Author summary

Impulsive choice behavior, or valuing immediate smaller rewards over larger, delayed rewards, is typically considered to be detrimental in decision-making. In this study, we use Markov Decision Processes (MDPs) to demonstrate that impulsive choices can be beneficial in three common decision-making tasks: Temporal Discounting, Information Sampling and an Explore-Exploit task. Specifically, we found that when a task environment is more uncertain than expected, an impulsive agent can collect more average reward than a non-impulsive agent. Our work suggests that impulsivity is not inherently negative. Valuing immediate rewards over delayed rewards can be an adaptive strategy when faced with uncertainty.

## Introduction

Impulsive decision making is frequently defined as disadvantageous. It has many descriptive definitions, including “choosing a smaller-sooner option when a larger-later option produces a better outcome,”[1] “swift action without forethought or conscious judgment,”[2] and “actions that are poorly conceived, prematurely expressed, unduly risky, or inappropriate to the situation and that often result in undesirable outcomes” [3]. Impulsivity is also considered a component of many clinical conditions, including gambling disorder and other behavioral addictions [4–6], substance-abuse [7–9], attention deficit/hyperactivity disorder [10,11], and other psychiatric disorders [2,12–14]. Taken together, these definitions and clinical manifestations suggest that favoring immediate rewards over delayed rewards leads to suboptimal outcomes [2,3,15–17]. Because impulsivity has carried this negative characterization, many studies have focused on impulsivity as maladaptive. However, there has been some investigation that suggests that impulsive choice behavior might be due to adaptation to the statistics of certain environments [18–22].

Impulsivity is measured with a variety of self-report questionnaires and laboratory tasks in human and animal subjects (For a review, see [23]). There are roughly 25 commonly used self-report questionnaires that measure impulsivity [15,24–27]. Laboratory tasks have also been designed to assess several dimensions of impulsivity, including motor impulsivity (for a review see [28]), attention impulsivity [29–31], risk preference [32–35], and impulsive choice behavior [36]. Choice impulsivity tasks, which we consider in the present manuscript, were developed to assess the weighting of immediate vs. future rewards. One commonly used choice task is Temporal Discounting [37–39], which measures preference for a smaller immediate reward or a larger future reward. Impulsive participants, by definition, favor the smaller, immediate rewards over the delayed, larger rewards. Information sampling tasks, such as the Beads task, are also used to measure the tradeoff between collecting more information or committing to a choice [40–45]. And N-armed bandit tasks that periodically introduce novel options have been used to assess the tendency for subjects to explore new options versus exploiting known options [21,46–50].

**Fig 1:**
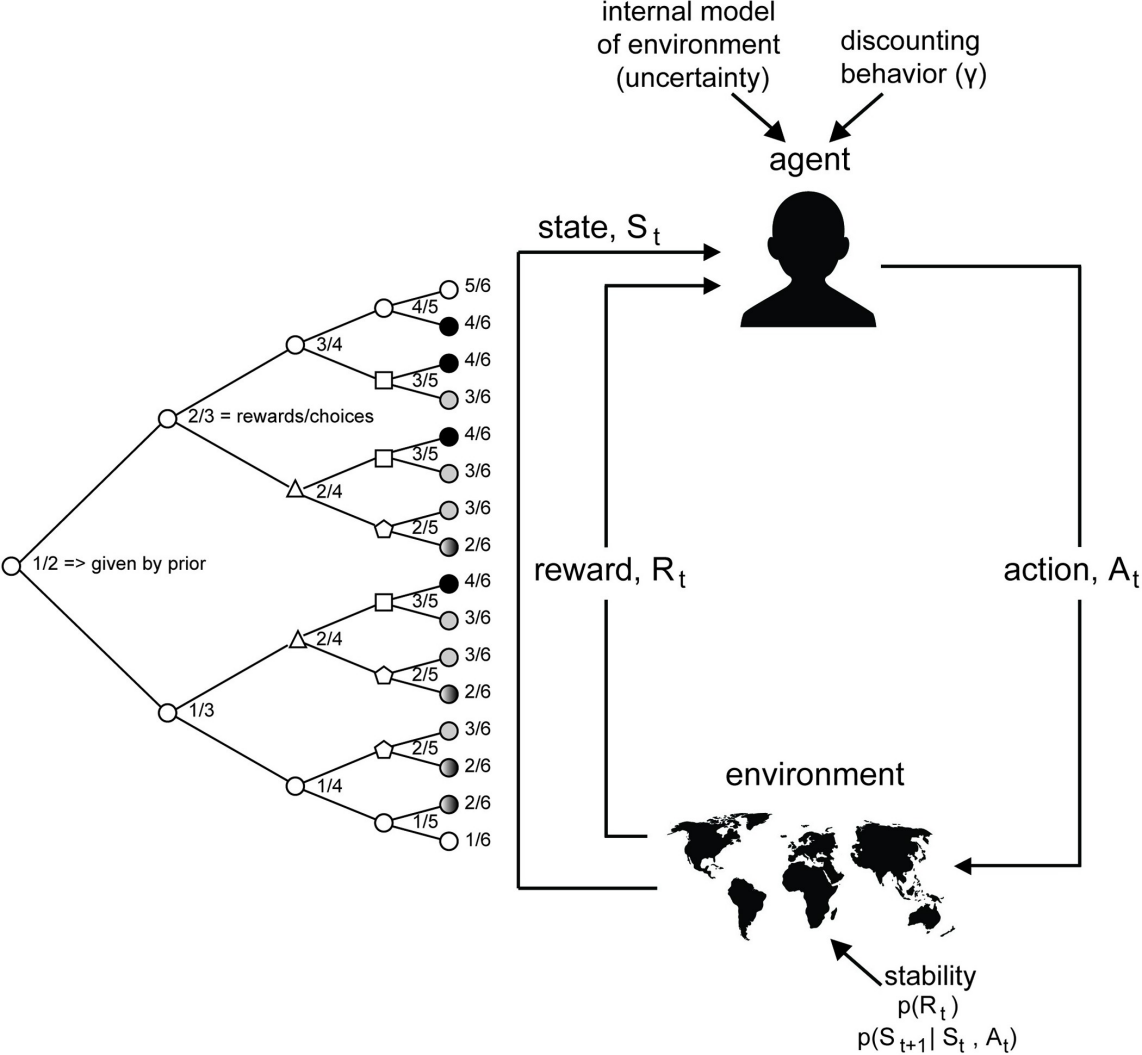
Agent and Environment Interactions in Reinforcement Learning (RL), Markov Decision Process (MDP) framework. A schematic of how an agent interacts with the environment and learns to maximize rewards in a MDP framework. An agent selects actions, A_t_, which lead to changes in state, S_t_ and rewards, R_t_, where t indicates the trial or time point. The agent’s internal model of the environment and weighting of future rewards, or discount factor, *γ*, affect the actions taken. The stability of the environment is captured by transition probabilities to future states p (S_t+1_|S_t_, A_t_) as well as the probability of receiving reward p (R_t_); these also affect reward outcomes. An example reward distribution tree for a binomial bandit is shown on the left for a bandit option in a choice task. As an agent selects the option that gives a probabilistic reward, it traverses the tree based on outcomes. Each node in the tree represents a choice point where that option was chosen. The node shape and shading indicate whether a node represents a unique state. Circle nodes are unique. Other shapes or shading of nodes indicate duplicate states that have multiple choice paths to them. While MDPs are independent of time and history, these factors often affect decision-making behavior. Each upper branch in the state space tree represents a rewarded choice, and each lower branch represents an unrewarded choice. Thus, the number at each node indicates the posterior over the number of rewards and the number of times the option has been chosen. Traversal through this tree leads to the accumulation of evidence for whether an option is highly rewarding or not rewarding, which in turn affects the agent’s future actions. Image credit: Wikimedia commons (bust image); Openclipart.org (map image).

In this paper, we used a Markov decision process (MDP) framework to compare the behavior of impulsive and non-impulsive agents in three common decision-making tasks where current choices affect future rewards. The MDP framework models decisions of an agent in an environment where the current state affects the immediate reward an agent can obtain, as well as the probabilities of transitioning to future states [51,52] (**Fig 1**). If it is assumed that the agent is maximizing the expected reward, the MDP provides insight into the optimal strategy (that is, to maximize over state-action values), in a decision-making task.

Within the MDP framework, action values, *Q*(*s*_*t*_, *a*), are the sum of immediate and discounted future expected rewards:

$$Q(s_{t},a)=r(s_{t},a)+\gamma \sum_{j\in S}p(j|s_{t},a)u_{t+1}(j)$$

where the first term *r*(*s*_*t*_, *a*) is the immediate expected reward in state *s* at time *t* if action *a* is taken, and the second term, $\gamma \sum_{j\in S}p(js_{t},a)u_{t+1}(j)$, estimates the discounted future expected value (FEV) of rewards. The second term, therefore, quantifies the future values of actions taken in the present, i.e. delayed rewards. This second term is the product of the discount factor, *γ*, and an expectation over future utilities, *u*_*t*+1_(*j*), with the expectation taken over the transition function, which is the conditional distribution of futures states, *p*(*j*|*s*_*t*_, *a*). Thus, the equation can also be framed as:

$$Q(s_{t},a)=IEV+\gamma *FEV$$

where IEV is the immediate expected value and FEV is the future expected value. For the (mostly) episodic tasks we will consider, the maximum average reward per episode would be obtained by an agent with a discount factor, *γ*, of 1.0 and the transition function given by the environment or the task. Algorithmically, discount factors are important for fitting infinite horizon models [53] but play a smaller role in fitting finite horizon, episodic models, unless episodes are very long. Discount factors are traits of agents, artificial or biological, and are not part of the environment. Naturally, if the discount factor, *γ*, is low, then the FEV affects the action value less.

Here we demonstrate parameter regimes where impulsive agents can perform better than non-impulsive agents; this effect is strongest when there is a mismatch between the agent’s expectation and the environment. In laboratory experiments, the question becomes whether reductions in weighted FEV occur due to a change in discount factor (*γ*) or due to a change in the transition function ($\sum_{j\in S}p(j|s_{t},a)u_{t+1}(j)$). The transition function is not always given (e.g. in temporal discounting tasks), or, when it is given, it may not be accurately approximated by subjects [54], and this misestimation can be mathematically indistinguishable from a change in discount factor. For example, participants may assume that environments are less predictable than is suggested by the experimenter (i.e. that the entropy of *p*(*j*|*s*_*t*_, *a*) is higher than stated), because participants have adapted to unstable environments outside the lab. This could result in an overall adjustment of discounting through the discount factor or flattening of the probability distribution affecting transitions to future states. In either case, the FEV is reduced, and the participant is more likely to choose immediate rewards. More formally, in unpredictable environments the conditional distribution, *p*(*j*|*s*_*t*_, *a*), has higher entropy, meaning that one cannot make choices that lead to desired future states, *j*. If some future states are rewarding and some are not, unpredictability means that the expectation over future utilities, $\sum_{j\in S}p(j|s_{t},a)u_{t+1}(j)$ will be smaller, or even negative. Because the value of delayed rewards is the product of the discount factor and the expectation over future utilities, subjects that do not value delayed rewards may be doing so because they have a lower discount factor, or because they assume environments have unpredictable transition functions. In laboratory experiments this is usually assumed to load on the discount factor, but these effects can also be captured by increasing the uncertainty of the transition function [43]. In this manuscript, we demonstrate that when the discount factor is low, this reduces the impact of the FEV and any related uncertainty caused by changes in *p*(*j*|*s*_*t*_, *a*). In cases where *p*(*j*|*s*_*t*_, *a*) is lower than expected, and future rewards are less likely, an impulsive agent can fare better than a non-impulsive agent.

In the present study we examined the tradeoff between the discount factor and uncertainty in three decision-making tasks that can be related to each other through the discount factor and MDP framework. We show that when task environments are more uncertain than an MDP model expects, agents with smaller discount factors outperform agents with higher discount factors in tasks where discount factors of 1 would be optimal if the transition function was accurately approximated by the agent. This correspondingly implies that agents, and possibly human subjects, that are adapted to relatively uncertain environments can outperform agents not adapted to uncertainty. While this second point follows directly from the models, it leads to an interpretation of impulsive choice strategies as optimal adaptations to environments with substantial uncertainty, rather than pathological deficits in decision making.

## Results

The goal of this study was to examine the hypothesis that impulsive choice strategies, defined as a relative preference for immediate over future rewards through the discount factor, can perform better than non-impulsive choice strategies, when environments are more uncertain than expected. More specifically, when agents are not able to make choices that lead to preferred future states, due to environmental variability, choice strategies that favor immediate rewards can be superior. We combined models of three decision-making tasks: Temporal Discounting, Beads, and Explore-Exploit into a single MDP framework and related the tasks to each other through the discount factor, which has been previously used to operationalize impulsive choice behavior [55,56]. In all three tasks, we dissociated the expectations of the agent from the true uncertainty in the environment, to establish the conditions under which an impulsive choice strategy would be beneficial. For each task, we varied the parameters to simulate uncertain and certain environments, to test whether impulsive and non-impulsive agents would fare better. In the certain environments, future rewards were more likely than agent’s expectations, and in the uncertain environments, less likely. To model impulsive and non-impulsive agents, we varied the discount factor, which captures the value of future rewards, and computed action values in the model. Thus, impulsive agents have lower discount factors (*γ*_*Impulsive*_) and weight immediate rewards more, and non-impulsive agents have higher discount factors (*γ*_*Non−Impulsive*_) and weight future rewards relatively more than immediate rewards. Although the statistics assumed by the agent vs. those that characterize the environment can be dissociated, only agents have discount factors.

In the Temporal Discounting task, the agents were given pairs of options with varying reward magnitudes and delays. Without manipulation of the future reward probability, the agent with the higher discount factor (i.e. less discounting) will collect more reward for choosing the larger, delayed rewards. However, we demonstrate that when the future reward is more uncertain than expected, the impulsive agent collects more average reward. In the Information Sampling task, the impulsive and non-impulsive agents are given bead draw sequences that are more or less informative about the majority color than expected. We demonstrate that when the bead information is less informative than expected, the impulsive agent collects more average reward by avoiding excessive draw costs for low value information. In the Explore-Exploit Task, the impulsive and non-impulsive agents choose between three bandits to learn which is the most rewarding option. Periodically, one of the bandits is replaced with a novel bandit. We demonstrate that when the substitution rate is high, the impulsive agent collects more average reward by not exploring the novel options. Thus, across three decision-making tasks, we show that when future rewards are more uncertain than expected, impulsive choices can lead to more reward.

### Impulsive agents benefit from choosing immediate rewards in a Temporal Discounting task

**Fig 2:**
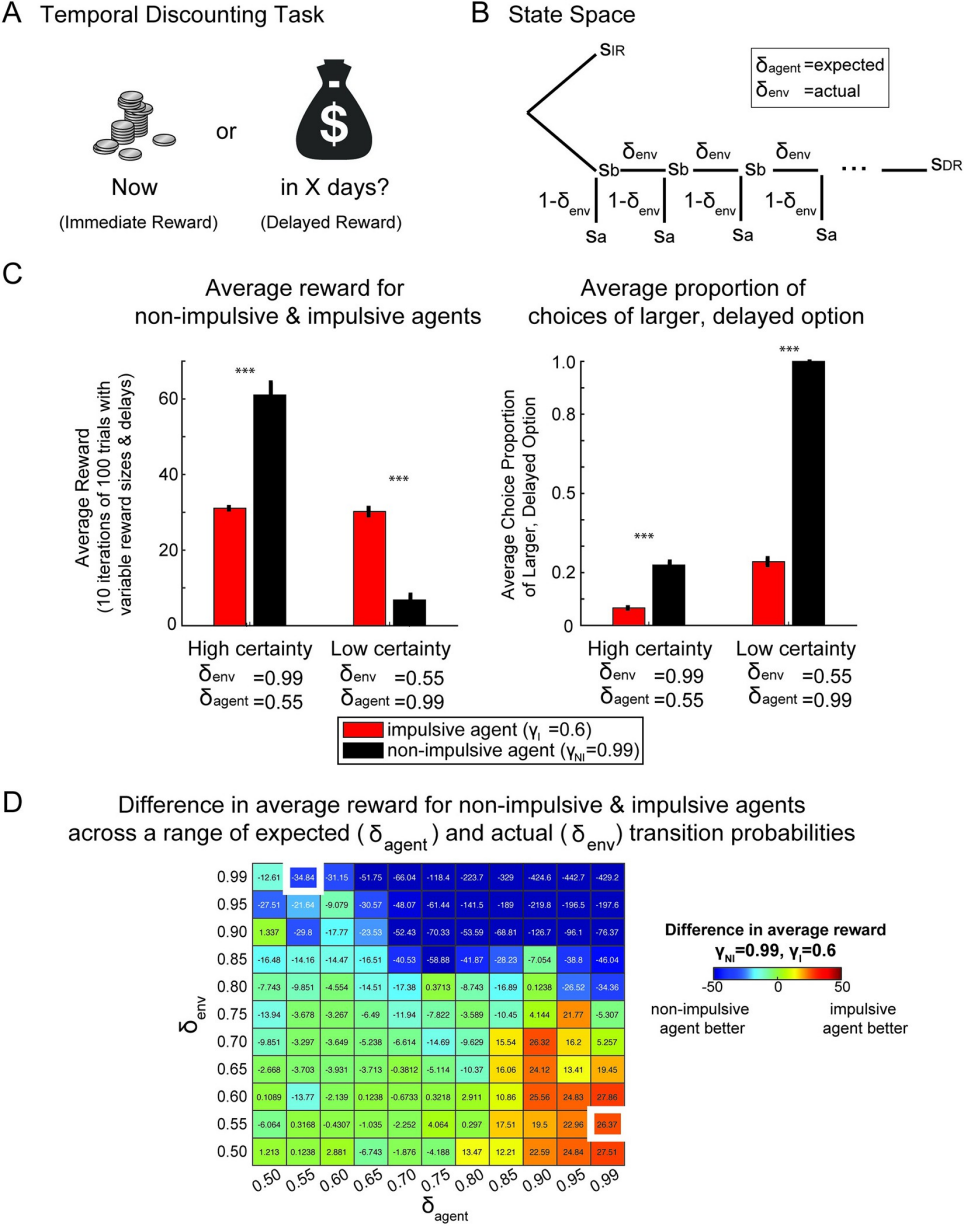
Temporal Discounting task and performance of impulsive and non-impulsive agents in different task environments. **A)** Task schematic for the Temporal Discounting task. Participants or agents are given a series of questions with two offers, one for a small immediate reward and the other for a larger, delayed reward. **B)** The state space tree for one pair of options in the task. The agent starts on the far left with a choice between the immediate reward or delayed reward. If the immediate reward is chosen, the agent proceeds on the upper branch to the immediate reward state (s_IR_) and always collects the immediate reward. If the agent chooses the delayed reward, the agent proceeds through the lower branch towards the delayed reward state (s_DR_). Along this branch are a sequence of intermediate transition states (s_b_) which the agent progresses through with probability δ. At each transition state, the agent might proceed to a terminal, non-rewarding state (s_a_) with probability 1-δ. The number of transition states is defined by the delay to the larger reward. **C)** Average reward collected and choice behavior across simulated trials in certain and uncertain task environments for impulsive and non-impulsive agents in the Temporal Discounting task. “High certainty” is when δ_*env*_>δ_*agent*_ and “low certainty” is when δ_*agent*_<δ_*env*_. The non-impulsive agent (black) has a discount factor of γ = 0.99 and the impulsive agent (red) has a discount factor of γ = 0.6. Left: Average reward collected for the two agents. Right: Average proportion of trials in which an agent selected the larger, delayed option. Error bars are s.e.m. across 10 iterations of 100 trials using variable reward sizes and delays. *** indicates p<0.0001 paired t-test. **D)** Difference in average reward across a range of δ_*env*_ and δ_*agent*_ values. The heatmap shows domains where the non-impulsive agent performs better (more blue), the impulsive agent performs better (more red) or there are marginal differences between the two agents (red). The values shown in each box on the heatmap is the difference in average reward for the two agents. The white boxes indicate the task regimes shown in Fig 2C. See S1 Fig for other discount factors. Image credit: Openclipart.org (coins image, money image).

The Temporal Discounting task was based on the Kirby delayed discounting questionnaire, which is typically used to evaluate how human participants value immediate and delayed rewards [38,43,57,58]. In this task and similar temporal discounting tasks, participants are presented with a set of choices between smaller immediate monetary rewards and larger, delayed monetary rewards (**Fig 2A**). Previous work has shown that delayed rewards are typically valued less than immediate rewards of the same size. However, it remains unclear why future rewards are discounted, and there exist multiple possible mechanisms [59]. Here we examined the performance of impulsive (low discount factor, *γ* = 0.6) and non-impulsive (high discount factor, *γ* = 0.99) agents that also assumed different state-transition probabilities to future rewards. We examined the performance of these agents in environments where the actual transitions to future rewards were stochastic, such that future rewards were not always collected. The link between uncertainty and the performance of impulsive and non-impulsive agents is straightforward in this task. However, it illustrates the point that we generalize in subsequent tasks.

The state space for this task consists of two branches, one representing the smaller, immediate reward, and the other representing the larger, delayed reward (**Fig 2B**). If the immediate reward is chosen, then progression to the terminal, rewarding state, *s*_*IR*_ is guaranteed and the reward is collected. If the delayed reward is chosen, the agent proceeds through a sequence of states representing the passage of time. The agent progresses through transition states (*s*_*b*_) towards the final delayed reward state (*s*_*DR*_) with probability δ or terminates at intermediate, non-rewarding states (*s*_*a*_) with probability 1−δ at each intermediate timestep. The transition states represent the passage of time and uncertainty about the delayed reward. The only decision is whether to take the immediate reward, or to pursue the future, larger reward.

In the model, the future expected value (FEV) of the delayed option, from the initial choice state, is calculated by discretizing the delay to the larger reward into steps with a probability of transitioning to each step with δ_*env*_. When the transition probability, δ_*env*_, to the delayed reward is high, the FEV of the delayed option is higher than the immediate reward. On the contrary, when the transition probability is low, the FEV of the delayed option is small.

Expanding upon the idea that in some cases, the value of the immediate option can be larger than the FEV of the delayed option, we examined whether an agent that discounted future rewards (i.e. impulsive) might fare better on average when the certainty of the delayed reward in the environment was worse than expected. In this case an agent expects a transition probability that is higher than the actual transition probability in the environment. We tested agents with two different discount factors (impulsive and non-impulsive) and two different transition probability assumptions, in two different environments. Specifically, we tested impulsive and non-impulsive agents under conditions in which the probability of transitioning to the delayed reward in the environment is higher than expected by the agent (δ_*env*_ = 0.99, δ_*agent*_ = 0.55) and under conditions in which the probability of transitioning to the delayed reward in the environment is lower than expected by the agent (δ_*env*_ = 0.55, δ_*agent*_ = 0.99). We simulated batches of trials with various sizes of rewards and delays. We then used the discount factor, γ, to model variable levels of discounting to reflect impulsive or non-impulsive behavior (γ_*I*_ and γ_*NI*_, respectively).

The results from testing these two agents in the high and low certainty environments shows that in the high certainty environment, the impulsive agent collects less average reward than the non-impulsive agent (**Fig 2C left**; paired sample t-test, t(9) = -20.92, p<0.001, d = -3.66, power > 0.99). In the low certainty environment, the impulsive agent fares better than the non-impulsive agent, by collecting more average reward (paired sample t-test, t(9) = 12.84, p<0.001, d = 6.06, power > 0.99). This outcome is driven by the frequency with which each agent selects the larger, delayed option in each environment. In both environments, the non-impulsive agent selects the larger, delayed option more often (**Fig 2C, right**). In the high certainty environment, when the transition probability is higher than expected (δ_*agent*_ = 0.55, δ_*env*_ = 0.99), the non-impulsive agent selects the delayed option more than the impulsive agent, due to the higher discount factor of the agent (paired sample t-test, t(9) = -21.10, p<0.001, d = -5.76, power > 0.99). However, the non-impulsive agent only selects the larger, delayed reward, about 30% of the time, due to the expectation of a low transition probability to delated rewards, as δ_*agent*_ = 0.55. In the low certainty environment (δ_*agent*_ = 0.99, δ_*env*_ = 0.55), the non-impulsive agent selects the delayed option every time and significantly more than the impulsive agent (paired sample t-test, t(9) = -52.25, p<0.001, d = -27.91, power > 0.99), due to the expectation of a high transition probability to the delayed reward. The impulsive agent selects the delayed option less often in both environments. There are combinations of transition probabilities for which the impulsive agent collects more reward, less reward, or roughly equal reward to the non-impulsive agent (**Fig 2D**). In general, when δ_*agent*_<δ_*env*_, the non-impulsive agent collects more average reward and when δ_*agent*_>δ_*env*_, the impulsive agent collects more average reward. When δ_*agent*_ and δ_*env*_ are both high (approximately > 0.8), the non-impulsive agent collects more average reward. Note that when both δ_*agent*_ and δ_*env*_ are very low (i.e. 0.55 and 0.5), the impulsive agent can collect at least as much or marginally more reward than the non-impulsive agent, showing that the main effects are driven by the mismatch between expected transition probability and actual transition probability. Furthermore, the effect sizes between the pairs of agents decrease as γ_*I*_ becomes closer to γ_*NI*_, as expected, but these relationships between δ_*agent*_, δ_*env*_, and reward remain the same (**S1 Fig**). Power analyses were conducted to make recommendations for an experiment with human subjects. Assuming an allocation ratio of 1.0 (i.e. equal number of subjects for each group), minimum power of 0.8, and alpha of 0.05, an experimenter would only need 3 participants with 100 completed trials, in each group to find a significant difference in average reward collected. However, given that the variability of human participants would be higher than that of our simulated agent behavior derived with a single discount factor, this is a low estimate of the number of subjects needed to run an experiment and see effects. For smaller effects in mean reward, (e.g. in the domain of δ_*agent*_<0.75 & δ_*env*_ = 0.65), power analyses suggest that an average of 200 participants would be required to detect statistical differences in mean reward.

In summary, choosing the immediate option in the Temporal Discounting task is advantageous when the larger, delayed reward is more uncertain than expected. This suggests that in a more complex task, it might be possible to find a regime where choosing immediate rewards is also beneficial. We discuss examples of such tasks in the following two sections.

### Impulsive agents benefit from guessing sooner in an Information Sampling (Beads) task

**Fig 3:**
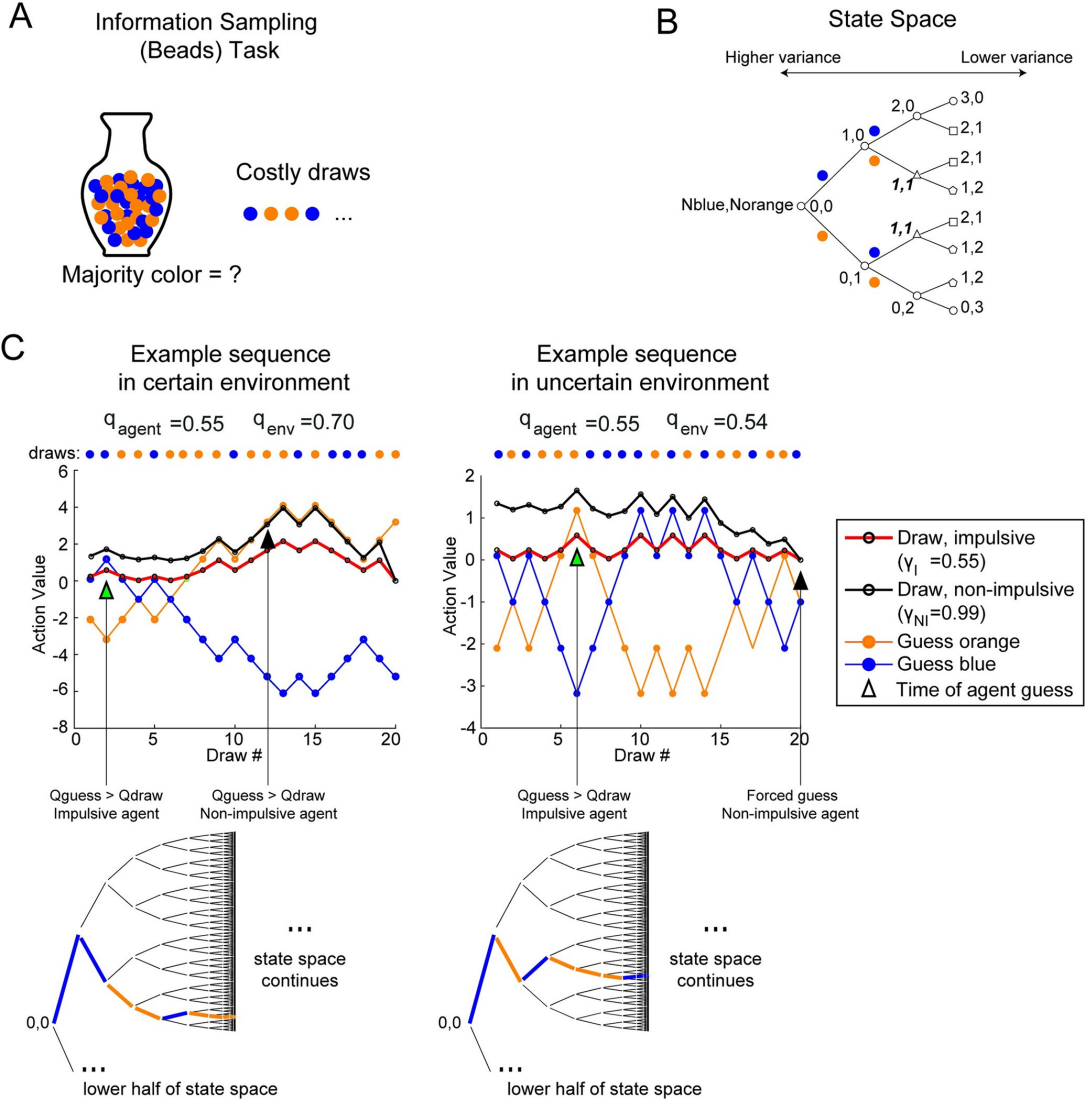
Information Sampling (Beads) Task and examples of agent performance in high and low certainty environments. **A)** Task schematic for the Beads task. In this task, the objective is to correctly guess the majority color of beads (e.g. orange or blue) in the urn. The participant or agent has the option to draw one bead at a time (for a cost, e.g. $0.10) to accumulate evidence. The agent’s goal is to accumulate sufficient evidence to make a confident guess without incurring maximum draw cost. Once the maximum number of draws is reached, the agent is forced to guess a color. An agent receives a reward for a correct guess (e.g. $10) or a cost for an incorrect guess (e.g. -$12). **B)** The state space tree for the beads task up to 3 draws. Each node represents the number of orange and blue beads that have been drawn thus far and a decision point, where the agent can either draw again, guess orange, or guess blue. If the agent draws another bead, they stochastically transition to the next state according to a binomial probability. At the start of the tree, the variance in the probability distribution over the majority probability is highest and decreases with increasing numbers of draws. Note that the states with the same number of orange and blue beads after 3 draws are the same state. We draw repeated states as separate for clarity,. Repeated states are illustrated by the shape of the node. Circular nodes are unique, nodes of other shapes indicate a repeated state. **C)** Two example bead draw sequences in certain and uncertain task environments and the behavior of impulsive and non-impulsive agents. On the left, a sequence of 20 draws is shown from a set of task parameters that creates an environment where there is high certainty the majority color is orange (q_agent_ = 0.55, q_env_ = 0.7, C_draw_ = 0.10, R_correct_ = 10, R_incorrect_ = -12). The plot shows the action values for guessing orange and guessing blue which are identical for both agents. The plot also shows the corresponding action values for drawing a bead for the non-impulsive agent (black) and the impulsive agent (red). Because the agents always select the largest action value on each time step, the agents only guess a color when the action value for guessing blue or orange surpasses the action value to draw another bead. In the case on the left, the non-impulsive agent guesses orange correctly after 11 draws (black arrow), whereas the impulsive agent guesses blue incorrectly after the first draw (green arrow). In the uncertain case (right), the task parameters create an environment where there is low certainty about the majority color (q_agent_ = 0.55, q_env_ = 0.54, C_draw_ = 0.10, R_correct_ = 10, R_incorrect_ = -12). The same traces for the action values are shown. The non-impulsive agent draws until it is forced to guess and incurs maximum draw cost (black arrow). The impulsive agent guesses correctly after 5 draws (green arrow). Below each plot of action values are the corresponding truncated state space trees, showing traversal through the state space for the example bead sequences. Only the top half of the state space tree is expanded through the first 10 bead draws.

In information sampling tasks, the objective is to collect information and make an informed decision based on accumulated evidence. We used the previously developed Beads task [41,44,45,60] to examine information sampling behavior. In the Beads task, the objective is to correctly guess the color of the majority of beads in an urn with two colors of beads (**Fig 3A**). To collect information about the proportions of colors, participants must draw one bead at a time, and incur a cost for each draw. Thus, at each step in the task, participants either choose to draw a bead or guess the majority bead color in the urn. If they guess correctly, they receive a reward (+10) and if they guess incorrectly, they receive a penalty (-12). This decision-making sequence can be represented with a state-space tree (**Fig 3B)**. In this diagram, each node represents a decision point to either draw a bead or guess the color of the urn. The state is given by the number of blue beads and the number of orange beads that have been drawn. At the start of the tree, (0,0), there are no beads of either color. As we proceed deeper into the tree, the variance of the binomial distribution over the proportion of beads of each color gets lower as we accumulate information through bead draws, and the estimates of the fraction of beads of each color is more accurate. If the urn fraction is low, e.g. 60%/40%, the uncertainty around the correct guess decreases slowly.

We hypothesized that an impulsive agent might fare better than a non-impulsive agent when the majority fraction of beads in the urn is lower than expected by the agent, and therefore bead draws are less informative than expected. To test this, we examined a condition in which the agents believed the majority color in the urn (q_agent_) was not far above chance (e.g. q_agent_ = 0.55). We then compared performance of impulsive (γ_*I*_ = 0.55) and non-impulsive (γ_*NI*_ = 0.99) agents in situations where the environment was more or less certain than expected (**Fig 3C**). The agent for this task has three actions available at each step: draw, guess blue, and guess orange, and at each step the agent picks the action with the highest value. In the more certain environment, the true underlying bead majority (q_env_) is 70% orange, and the action value for guessing orange continues to increase as draws are made and evidence accumulates that orange is the majority color (**Fig 3C, left**). When the action value for guessing one of the two colors surpasses the action value for drawing a bead, an agent will stop and guess that color. For the impulsive agent in the certain environment, the agent guesses a color after the first draw, which leads to an incorrect choice of blue. On the contrary, the action value for drawing a bead for the non-impulsive agent starts out high and the action value for guessing orange surpasses the action value for drawing only after 11 draws. The agent has accumulated evidence that the majority is likely orange and chooses orange correctly. The non-impulsive agent with the higher discount factor values future rewards and is driven to go further into the state space to reduce uncertainty about the majority color. The choice to guess a color terminates the sequence and therefore does not depend on the discount factor, as there are no future possible states that can be reached after a guess, and the discount factor only affects future state values.

On the other hand, in the uncertain environment, the action values for guessing each color do not diverge as clearly because subsequent draws are not consistently of one color (**Fig 3C, right**). In this example, the beads are sampled from an urn with 54% orange beads. This low majority drives the action value to draw a bead for the non-impulsive agent to stay higher than the action values for guessing the two colors until the max number of draws allowed, at which time the agent is forced to guess a color. In contrast, the impulsive agent makes multiple draws, but fewer than the non-impulsive agent, and guesses orange correctly. The impulsive agent was able to guess without accruing as much cost from the charges for additional bead draws. The partial state spaces for these examples show that the bead sequences start out identically for the first two draws of each of the bead sequences, but then the draws in the certain environment (left) quickly dive towards the lower edge of the subtree, reflecting increased probability of a majority color (**Fig 3C, bottom**). The path through the state space in the uncertain environment meanders towards the middle branches of the state space tree. On average, the closer to chance the majority color fraction, the less consistent the path through the state-space will be across trials of bead sequences.

**Fig 4:**
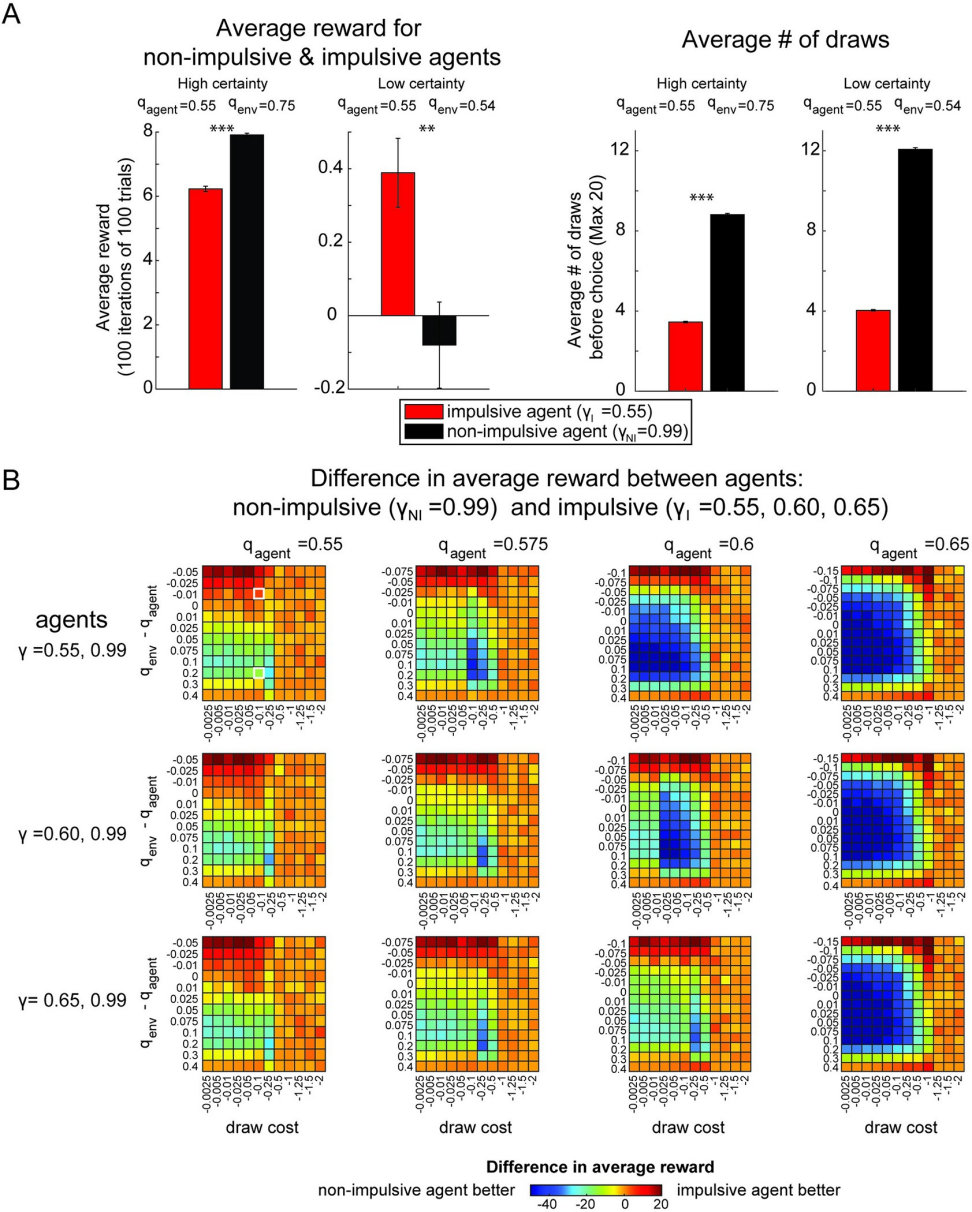
Performance and choice behavior of impulsive vs. non-impulsive agents in the Information Sampling (Beads) Task. **A) Left:** Average reward collected across simulated trials in certain and uncertain task environments for impulsive and non-impulsive agents in the Beads task. The non-impulsive agent (black) has a discount factor of γ = 0.99 and the impulsive agent (red) has a discount factor of γ = 0.55. Error bars are s.e.m. across 100 iterations of 100 trials using bead sequences from two different task parameters (q_env_ = 0.75 certain environment, q_env_ = 0.54 uncertain environment, q_agent_ = 0.55. C_draw_ = 0.1). **Right:** Average number of bead draws before guessing a color for each model in each task environment. In both task environments, the impulsive agent (red) draws similarly often, but significantly less than the non-impulsive agent (black). *** indicates p<0.0001 ** indicates p<0.001. **B)** Model performance across a range of parameter values. Each panel is a heatmap showing the differences in average reward for a pair of non-impulsive and impulsive agents, indicated by the discount factors on the far left. Each column has a set of heatmaps for the expected majority fraction of beads, q_agent_. Each row has a set of heatmaps for a pair of discount factors (impulsive & non-impulsive). The x-axis of each heatmap is the draw cost and the y-axis is the difference between the model input q_agent_ and the majority fraction used to generate the bead draws, q_env_. The color of the heatmap indicates whether the impulsive agent (red) or non-impulsive agent (black) collected more reward. More blue values indicate the non-impulsive agent collected more average reward and more red values indicate the impulsive agent collected more reward. As q_agent_ increases (left to right), the domain in which the non-impulsive agent performs better expands. The white boxes in the heatmap in the top left panel highlight the data used to create the bar plots Fig 4A (left). All heatmaps were generated using R_correct_ = 10, R_incorrect_ = -12. See S1 Fig for (B) with R_correct_ = 10, R_incorrect_ = -10.

To compare the average performance and choice behavior of the two agents in these environments, we simulated batches of bead sequences and choices using agents with the two discount factors. In the certain environment, where the majority fraction of beads is high, the non-impulsive agent with the higher discount factor (γ_*NI*_ = 0.99, black) collects more average reward (paired sample t-test, t(99) = -20.70, p<0.001, d = -2.93, power > 0.99) (**Fig 4A**). In the uncertain environment, the impulsive agent (γ_*I*_ = 0.55, red) collects more reward, despite both agents collecting less reward than in the certain environment (paired sample t-test, t(99) = 4.16, p<0.005, d = 0.59, power > 0.99). The reason for this is illustrated by the average number of draws each agent takes before guessing the color of the majority **(Fig 4A, right panel**). In both task environments, the non-impulsive agent makes more average draws before making a choice (paired sample t-test, t(99) = -104.76, p<0.001, d = -14.82, power > 0.99 for certain environment, t(99) = -139.74, p<0.001, d = -19.76, power > 0.99 for uncertain environment). This leads to a more informed choice in the certain environment, but in the uncertain environment, this only leads to a small improvement in guessing accuracy, and on average accrues more cost. The impulsive agent on the other hand, does not make as many draws before making a guess about the majority color, and thus avoids accruing additional draw costs for draws that do not improve the accuracy of the guess. The bead information in the uncertain environment is not only less reliable than expected, as the actual fraction of beads of one color (q_env_) is lower than what is expected by the agent (q_agent_), but also less informative, as the environment majority fraction (q_env_) is closer to 0.5. The bead information in the certain environment is also unreliable, in the sense that it does not reflect the expected majority fraction (q_agent_), but is more informative, as it provides a better estimate of the actual majority color.

Power analyses suggest that to observe these effects in an experiment with human subjects, (assuming minimum power of 0.8, alpha 0.05), a minimum of two subjects would be required to observe differences in choice behavior. However, approximately 20 participants in each group, impulsive and non-impulsive, would be the minimum number of subjects to observe differences in average reward collected in the uncertain environment as shown in Fig 4A. This number is a low estimate, as an experiment would have to account for the variability in discounting across subjects in the participant pools.

We also examined relative performance across a wider space of parameters, including the majority fraction of beads in the urn that is used to generate the bead draw sequences (q_env_), the agent’s belief about the majority fraction of beads (q_agent_), the draw cost (*C*_*draw*_), and the model discount factor (γ). We varied these parameters for multiple impulsive agents (γ = 0.55, 0.6, 065) and compared the average reward collected by the impulsive agents and the non-impulsive agents (γ = 0.99) across these task conditions (**Fig 4B**). As q_agent_ increases, the area of the parameter domain in which the non-impulsive agent fares better, expands. There exists a range of task parameters where an impulsive agent can collect more reward than a non-impulsive agent. For all task conditions, *R*_*correct*_ was +10 and *R*_*error*_ was -12 to encourage more than one draw from the impulsive agent. However, there also exist parameter domains where impulsive agents can perform better than non-impulsive agents when *R*_*correct*_ = |*R*_*error*_| (see **S2 Fig**). Thus, in an information sampling task, impulsive behavior can be beneficial when the information that is accumulated is less informative than expected and is associated with a growing cost.

### Impulsive agents benefit by exploring novel options less in an Explore-Exploit task

**Fig 5:**
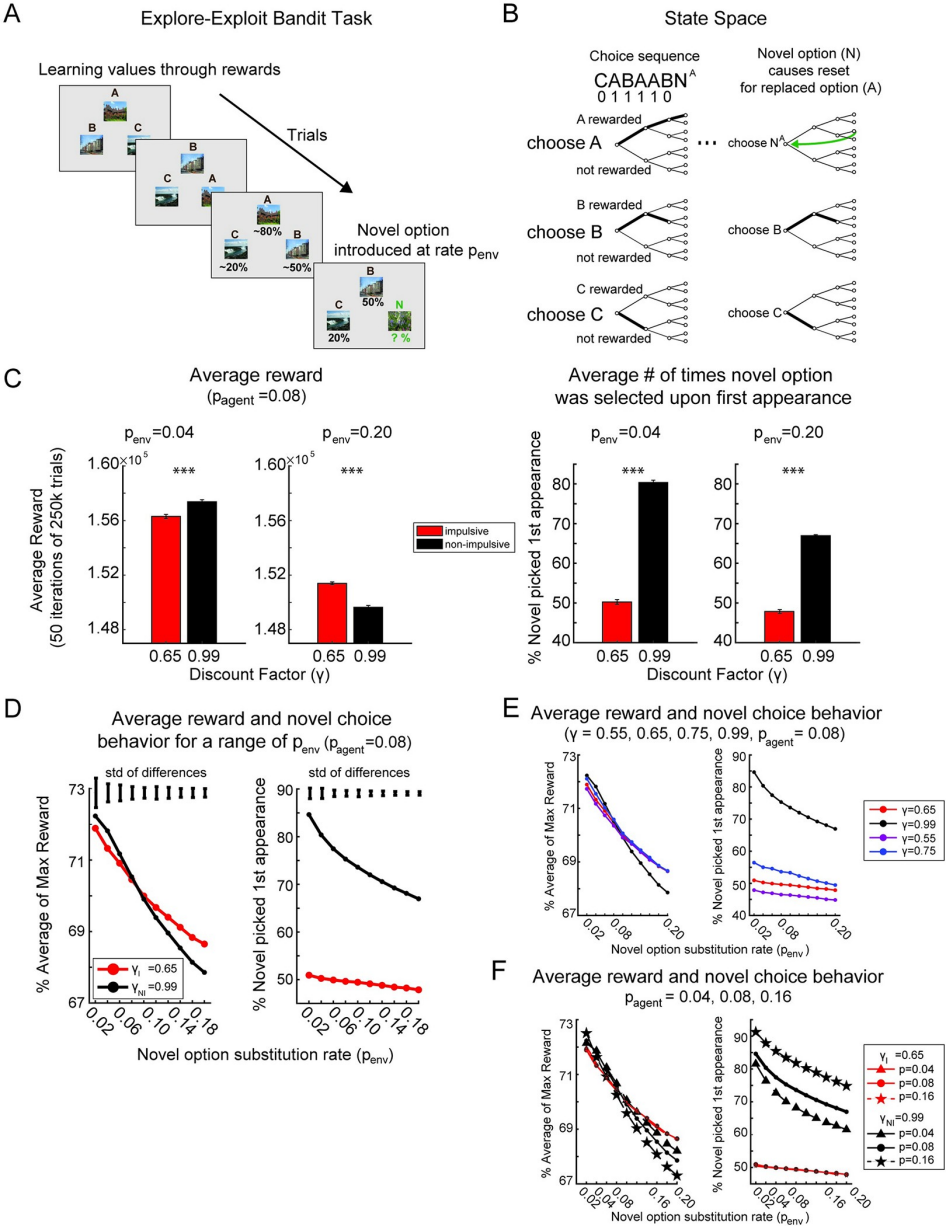
Explore-Exploit bandit task with novelty. **A)** Example sequence of trials in the Explore-Exploit task. In this example, each option (A, B, C) is a picture with an underlying reward rate. The agent or participant must learn the values of the three options through experience of choosing the options and receiving or not receiving a reward on each trial. In this example, the agent has learned the approximate values of the options over the course of multiple trials (not all are shown) and then a novel option is substituted for one of the options (option A). The novel option substitution rate (p_env_) affects the number of trials the agent has to learn about an option. When p_env_ is high, it is harder for the agent to learn about the underlying values of the options. **B)** State space representation of the Explore-Exploit task. Each option can be represented with a separate subtree. Thus, for an example sequence of choices such as C, A, B, A, A, B, the agent progresses through 1 step of the tree for C, three steps for A, and two steps for B. The agent progresses to an upper branch or lower branch depending on whether the choice was rewarded. Rewards are shown for this example sequence as 0s or 1s. Thus, the agent progresses through the uppermost branches of the subtree for option A, as it was rewarded all three times it was chosen. The introduction of a novel option causes the position in the subtree for that option to reset. When the novel option is presented at the end of this sequence and replaces option A, the agent jumps back to the start for that option, as reward history no longer represents the underlying value of the novel option. **C)** Bar plots of average raw reward (left) and average selection of the novel option upon first appearance (right) in low and high certainty environments. On the left, average reward for the non-impulsive (black) and impulsive (red) agents at p_env_ values of 0.04 and 0.20 are shown. On the right, average selection of the novel option upon first appearance is shown for the same values of p_env_. *** indicates p<0.0001. Error bars on plots s.e.m. across iterations. Error bars above plots represent the standard deviation of the differences between the mean values for the non-impulsive and impulsive agents. **D)** Average % of maximum possible reward and average novel option choice behavior across a range of novel option substitution rates for both the non-impulsive (black) and impulsive agent (red). On the left, the plot of average reward shows a that when the novel option substitution rate (p_env_) is low, the non-impulsive agent collects more reward than the impulsive agent, but when p_env_ is high (greater than 0.1), the impulsive agent collects more reward than the non-impulsive agent. The plot of novel choice behavior shows that for all novel option substitution rates tested, the non-impulsive agent selects the novel option significantly more often than the impulsive agent on the first trial it appears. Error bars above the graphs represent the standard deviation of the differences between the mean values for non-impulsive and impulsive agents. **E)** Average % of maximum reward and choice behavior for a range of discount factors and p_agent_ = 0.08. **F)** Average % of maximum reward and choice behavior for discount factors shown in (C) and (D) with p_agent_ = 0.04, 0.08, 0.16. Image credit: Wikimedia Commons (scene images).

In the Explore-Exploit task, there are three options that pay off with an equal, fixed reward, but with variable reward probabilities. An agent must learn which option is most valuable by selecting the options and experiencing reward. The bandits are stationary, in that the reward rate for each option remains fixed. However, novel choice options replace familiar options at stochastic intervals. When this happens the agent must choose between exploring the novel option, which has an expected value of 0.5 before it is sampled, and exploiting familiar options, for which the agent has an estimated reward probability (**Fig 5A**). In this example series of trials, three choices (A, B, and C) are shown. Through exploration of these options, the agent learns the approximate reward rate of each of the options, and should learn to pick A more often, as it is the most valuable. In the last panel in the series, a novel option is introduced to replace option A. The value of the novel option on the first trial is not known. The rate of replacing an option with a novel option is parametrized with the substitution rate of the environment (p_env_). The higher the substitution rate, the more volatile the environment. Substitution with novel options affects where an agent is in the state space.

The state space for this task can be represented with one binomial tree for each option. As an option is chosen, the agent traverses that particular tree towards the upper half of the tree if the option is rewarded, and lower half if the option is unrewarded. Introduction of novel options resets the tree for the option which was replaced to the root node. For example, consider an agent that selects among three options (A, B, and C) and makes a sequence of three choices: C, A, B, A, A, B. After these choices, a novel option is introduced to replace option A (**Fig 5B**). In this example, assume A was chosen 3 times and rewarded 3 times, then the position in the tree would be along the uppermost branch of the state space tree for option A. If B was chosen twice, and rewarded and then not, rewarded, the tree for option B would appear as shown. C was chosen once, and not rewarded, and the position in the state space would be one step along the lowermost branch. When a novel option is introduced, as in this case, after three choices for option A (N^A^), the agent’s position in the tree for option A jumps back to the start, because now nothing is known about the new option. The positions in the other choice trees (B and C) remain the same. As the substitution rate gets higher, the agent rarely reaches deep nodes in the tree, which reflects more accurate reward probability estimates, for any of the options, because they are replaced before the agent can reach an accurate estimate of the value of an option. We hypothesized that in this context, an agent that discounts future rewards might fare better by exploiting known options as long as possible, rather than exploring novel options. Exploring novel options is a time investment that only pays off, on average, in the future, and therefore exploration in this context is more valuable with higher time-constants, because the relative value of exploration is obtained in the future.

To test the hypothesis that an impulsive strategy would fare better than a non-impulsive strategy when the novel substitution rate was high, we varied the discount factor (*γ*_*I*_ = 0.65, *γ*_*NI*_ = 0.99) and novel option substitution rate (p_env_). Similar to the beads task, we examined a situation in which the agent believed the substitution rate (i.e. the probability) per trial was 0.08, and the actual substitution rate was above (0.2) or below (0.02) that value (i.e. p_agent_ = 0.08, p_env_ = 0.02 or 0.2). When the substitution rate is higher, the environment is more uncertain because options are frequently replaced, and when the substitution rate is lower the environment is less uncertain. In the case where the substitution rate in the environment was lower than the agent expected, the non-impulsive agent collected more average reward (**Fig 5C, left**) (paired sample t-test, t(49) = -13.74, p< 0.0001, d = -1.94, power > 0.99). In the case where the substitution rate in the environment was higher than the agent expected, the impulsive agent collected more average reward (**Fig 5C, left**) (paired sample t-test, t(49) = 47.74, p< 0.0001, d = 6.75, power > 0.99). This corresponded to a difference in choice behavior of the novel options. In both sets of task conditions, the impulsive agent selected the novel option less often (**Fig 5C, right**) (paired sample t-test, t(49) = -202.23, p<0.0001, d = -28.60, power > 0.99 for certain environment, t(49) = -379.45, p<0.0001, d = -53.66, power > 0.99 for uncertain environment). As the substitution rate increased, both agents selected the novel option less often.

The average reward collected, and novel choice behavior, differ between the impulsive and non-impulsive agent depending on the novel option substitution rate (**Fig 5D**). The impulsive agent collects less reward than the non-impulsive agent when the novel option substitution rate is lower than 0.06, and more than the non-impulsive agent when the substitution rate is higher than 0.14 (**Fig 5D, left**). Note that at 0.02, only 2 out of every 100 trials are novel option trials, and thus the agents perform similarly due to limited encounters with novel options. On average, the impulsive agent chooses the novel option less upon first appearance across all substitution rates (**Fig 5D, right**). A two-way ANOVA was performed to analyze the effect of discount factor (γ) and novel option substitution rate (p_env_) on average reward. As substitution rate increased, average reward decreased (main effect: substitution rate, F(9,980) = 2176.12, p<0.001). At low substitution rates less than 0.10, the non-impulsive agent (*γ*_*NI*_ = 0.99) collected more reward than the impulsive agent (*γ*_*I*_ = 0.65) and at high substitution rates, greater than 0.10, this effect reversed such that the impulsive agent collected more average reward than the non-impulsive agent (main effect: discount factor F(1,980) = 91.01, p<0.001, interaction: substitution rate x discount factor F(9,980) = 65.16, p<0.001). Similarly, a two-way ANOVA was performed to assess the effect of discount factor and substitution rate on novel choice behavior. As the substitution rate increased, selection of the novel choice option upon first appearance decreased for both agents (main effect: substitution rate, F(9,980) = 2298.43, p<0.0001). Furthermore, the non-impulsive agent selected the novel option significantly more often than the impulsive agent across all substitution rates (main effect: discount factor, F(1,980) = 316014.59, p<0.0001). A change in environment substitution rate had a larger effect on novel choice behavior for the non-impulsive agent, as the non-impulsive agent had a ~20% decrease in selection of the novel option from the lowest substitution rate (p_env_ = 0.02) to the highest substitution rate (p_env_ = 0.20) and the non-impulsive agent only selected the novel option 45–50% of the time across all substitution rates (interaction: substitution rate x discount factor, F(9,980) = 1170.38, p<0.0001).

We also examined relative performance across a range of agent substitution rates and discount factors. First, we varied the impulsive agent discount factor (*γ*_*I*_) while keeping the agent’s substitution rate, p_agent_ = 0.08, constant (**Fig 5E**). As the discount factor of the impulsive agent (*γ*_*I*_) became closer to the discount factor of the non-impulsive agent *γ*_*NI*_, the differences between the agents across a range of p_env_ decreased. Next, we varied the agent’s substitution rate (p_agent_) while keeping the discount factors constant (*γ*_*I*_ = 0.65, *γ*_*NI*_ = 0.99) (**Fig 5F**). Changing the agent’s substitution rate had negligible effects on average reward and novel choice behavior for the impulsive agent, however, changing p_agent_ for the non-impulsive agent affected both average reward collected and novel option choice behavior (Fig 5F). When the trained substitution rate was the highest (p_agent_ = 0.16), the non-impulsive agent collected the least average reward when p_env_ was greater than 0.10. These results suggest that the discount factor has a larger effect on choice behavior than the trained substitution rate. In all cases, there were no differences in average reward between the impulsive and non-impulsive agents at the agents’ trained substitution rates. Thus, it was the differences in discount factors and mismatch between expected substitution rate by the agent (p_agent_) and the actual substitution rate (p_env_) that were responsible for differences in average reward and choice behavior between impulsive and non-impulsive agents.

Power analyses showed that to observe effects like those shown in Fig 5C, only 7 iterations would be required to observe the smallest effect. However, each of these iterations included 250,000 trials. To provide guidance for readers with regards to effect sizes that might be observed in experiments using human participants, we ran simulations using a more reasonable number of trials per iteration that might be possible in an experiment, while keeping the number of iterations, 50, fixed. Simulations with 5000 trials for each iteration (or theoretical human participant) produced results that were still significant for some substitution rates, but much weaker and not over the entire range of substitution rates (two-way ANOVA, main effect: substitution rate F(9,980) = 1.11, p = 0.2919, main effect: discount factor F(9,980) = 45.46, p <0.0001, interaction substitution rate x discount factor F(9,980) = 2.36, p < 0.05). In particular, following the example in Fig 5C, at p_sub_ = 0.04 differences in mean reward between agents were not statistically significant (paired sample t-test, t(49) = -0.79, p = 0.43, d = -0.11) but differences at p_sub_ = 0.20 remained significant (paired sample t-test, t(49) = 6.58, p<0.0001, d = 0.93). This is because with only 5,000 total trials, p_sub_ = 0.04 results in only 200 novel option trials. Thus, if someone were interested in pursuing an experiment with human subjects, it would be possible to titrate the number of trials with available participants in each subject group to observe benefits of impulsive behavior at high substitution rates.

In summary, we have shown that across three common decision-making tasks, that non-impulsive choice strategies can be beneficial. In particular, this is true when task variables create an environment where future rewards are less certain than expected.

## Discussion

We used Markov decision process models to examine the trade-off between environmental uncertainty and the advantages of impulsive choice strategies. We found, across three tasks, that when the environment was more uncertain than expected, agents with impulsive choice strategies that favored immediate over future rewards were more effective than agents with less impulsive choice strategies. In Temporal Discounting, an agent that selects an immediate, smaller, certain option, earns more rewards than an agent that selects future, larger, uncertain options. This finding extends to other tasks that have been used to measure impulsivity. In the Information Sampling task, when subjects draw beads (at a cost) to improve their ability to guess the correct urn color, deciding early is advantageous when beads are less informative than expected. This is particularly true when incorrect choices lead to large losses. Finally, in an Explore-Exploit task in which novel options are periodically introduced, exploration of novel options is only beneficial when they will be available for exploitation in the future. Therefore, when the available options turn over more frequently than expected, exploration is less valuable and impulsive strategies that select options with higher immediate expected values are more advantageous. Our results show that an impulsive choice strategy, which is often considered maladaptive, can be advantageous when environments are consistently more uncertain than expected.

The value of future rewards depends on the ability of an agent to execute a sequence of choices that lead to future states that deliver those rewards. They also rely on the subjective weighting of future rewards. When environments are uncertain, actions will not necessarily lead to desired future states. This leads to decreased future expected values (FEV) and increased relative value of immediate reward. If the conditional distribution of future states, *p*(*j*|*s*_*t*_, *a*), is broad (i.e. high entropy), conditioned on actions and states, an agent cannot control its transition to a future state because many states are likely to occur. Stated another way, agents have limited ability to control future outcomes. If only a few future states have high utility, and particularly if some futures states have negative utility, this lack of control will significantly decrease the values of the future expected reward term in the action value equation. Thus, agents that are adapted to uncertain environments should learn to consistently reduce future expected values. Here, we have simulated this reduction by manipulating the discount factor in situations where the agent had a different expectation for *p*(*j*|*s*_*t*_, *a*) than given by the task and showed that having a low discount factor can be beneficial.

Laboratory decision-making tasks used to measure impulsivity assess subjects under the assumption that all subjects will assume the same transition probabilities, which are often given by task instructions, or left implicit. If, however, participants have adapted to different levels of uncertainty in the environments in which they live, they may make choices with different implicit levels of uncertainty in the distributions of conditional state transitions. As these are assumed fixed by experiments, differences in behavior will be attributed to differences in discount factors. However, it is also possible that subjects have poor estimates of transition probabilities, and it is not straightforward to dissociate an agent’s discount factor from the uncertainty in the state transition function they bring to a task, and both can decrease future expected values. In other words, task performance is always optimal when the statistics of the environment are accurately modeled. However, if an agent has different expectations than the true environment statistics, as was the case in this study, then discounting future rewards can be beneficial to task performance. We chose to model situations in which the transition probabilities of the environment were more uncertain than expected by the agent, because this led to an advantage for smaller discount factors. However, we could also have matched the discount factors, and shown that in uncertain environments, agents that better approximated that uncertainty would do better than agents that thought the environment was more certain. Either reducing the discount factor or increasing estimates of environmental uncertainty decreases the value of future rewards, and therefore makes immediate rewards relatively more valuable.

The Temporal Discounting task in our study was modeled after the KDD behavioral assessment, which is a questionnaire used to assess subject specific preferences for smaller, immediate rewards relative to larger, future rewards [58]. We simulated stochastic environments, such that future rewards were not always delivered. Importantly, we modeled the delay to the larger reward with a transition probability and used the MDP, a utility based model, to compute action values when the transition probabilities were higher than expected (certain environment) and lower than expected (uncertain environment), which made the TD simulations risky intertemporal choices. We found that impulsive agents performed worse when transition probabilities to larger, delayed rewards were higher than expected, similar to previous findings using probabilistic future rewards (for reviews, see [61,62]). However, when the transition probabilities to delayed rewards in the environment were lower than expected, the impulsive agent with the lower discount factor collected more average reward than the non-impulsive agent that chose larger, future rewards that were not delivered. The success of the impulsive agent was amplified by the mismatch between the expectation about future reward and the underlying probability of reaching that reward. As previously described, impulsivity is frequently given a negative interpretation. In contrast, we demonstrate that choosing a smaller, immediate reward can be beneficial in some cases, in this case, risky intertemporal choice. It remains an open debate whether attribute-comparison (i.e. time vs. time and probability vs. probability) or utility based models are more appropriate for capturing intertermporal choice behavior and neural representation, and there are many kinds of intertemporal choices based on combinations of attributes [62]. Here we demonstrate an example where an impulsive agent can perform better than a non-impulsive agent, and this example could be extended to other kinds of intertemporal choices by using mismatched expectations across a range of attributes. Recent work with related discounting tasks used to assess weighting of immediate and future rewards, such as the Marshmallow Task [63], have also shown that preference for immediate rewards can be related to the perceived reliability of the experimenters, and trust, rather than trait impulsivity, which suggests that the accuracy of expectations can affect choice behavior [64]. Other work has suggested that immediate choices in the Marshmallow task are rational adaptation to time delays rather than failures of self-control [20]. Thus, although patients with substance use disorders and some psychiatric disorders can exhibit higher impulsive choice in behavioral tasks [65], and this is given as a possible dimensional explanation of their disorder, favoring immediate, smaller rewards, can be beneficial when the task environment makes future rewards less likely than expected.

Information sampling tasks have also been used to assess impulsivity [5,42,66]. Variations on these tasks include random dot motion perceptual inference [67], perceptual-motor inference [68], and sequential sampling paradigms [44,66,69–71]. We modeled choices in the Beads task, which has also been used to assess discrete information sampling with sampling cost [41,44,45]. In this task, participants are asked to guess the majority color of beads in an urn. In each trial, they can draw an additional bead from the urn for a small cost or guess the majority color. Drawing additional beads, therefore, improves accuracy, but at a cost. Past work has used a variety of models to capture both reaction time and choice behavior in perceptual inference tasks, including the well-known drift diffusion framework [72,73] and variants [67,74], including full POMDP developments [67], similar to what we have used. The drift diffusion framework captures the decision to terminate information sampling with a threshold crossing. Here we modeled the decision without the need to fit a threshold by quantifying the action values for continuing to accrue information (i.e. draw a bead) versus making a choice based on previously gathered information (i.e. guessing a color)[41,44]. We manipulated the probability distribution for the actual bead draws such that they were higher or lower than the majority fraction the agent expected. We also made the cost for guessing incorrectly larger than the cost for guessing correctly, to encourage drawing behavior from the impulsive agent. When the majority fraction of beads in the urn was lower than the agent’s expectation, the impulsive agent accumulated more reward than the non-impulsive agent, because the non-impulsive agent accumulated costs for draws that were less informative than expected.

We showed that an impulsive agent can perform better in conditions in which we manipulated cost and uncertainty, but the effect is strengthened when the cost for guessing incorrectly is larger than the reward for guessing incorrectly. There has been some past work with the Beads task and asymmetric reward structure, but to our knowledge, only small and large rewards, not cost for guessing [75]. It would be interesting to explore asymmetric payouts in future work. Based on previous modeling of cognitive resources during information sampling in the Beads task, we would predict that a loss context would inhibit guessing for human participants in a way that reflects general risk preference, rather than a precise, online computation that would be cognitively demanding [76]. Past work has also shown that manipulating sampling costs can lead to changes in sampling, such that participants can be driven to oversample when sampling costs are low [68,77]. Sampling can also be affected by perseverative behaviors, not just information seeking, particularly in impulsive subjects. In one study, subjects were asked to report their estimate of the probability of the majority color in a variant of the Beads task, and subsequent analyses showed that schizophrenic patients, characterized with impulsive behavior, had persistent drawing that correlated with the frequency of clinical delusions. However, when delusions were controlled for in analyses, the same patients exhibited decreased information seeking compared to healthy individuals, suggesting that perseverative drawing is sometimes unrelated to the goal of information seeking [78].

Our results show that not only the cost to sample, but also the expected utility of the information sampled, can affect sampling and overall performance. However, the simulations here do not account for perseverative actions, which can be a feature of impulsivity and drive what appears to be perseverative information seeking. In our simulations, the impulsive agent benefitted from sampling less when the information gained from sampling was less informative. Future experiments involving impulsive human subjects could test both the effects of this loss context and also incorporate a separate term in the model for perseverative drawing that is independent from drawing related to information seeking.

Impulsive choice has also been shown to be related to novelty-seeking in clinical disorders and substance abuse [79–83]. However, these studies frequently use self-report questionnaires that measure sensation seeking as a metric for novelty seeking behavior. Our measure of novelty seeking is related to the explore-exploit trade-off, and operationalizes an investment in learning about a novel option (i.e. exploration) because the investment may pay off in the future (exploitation), in a well-characterized bandit task with novel options [44,46,84–86]. In the Explore-Exploit task, we manipulated the substitution rate of novel options. When the substitution rate was higher than expected, the impulsive agent collected more reward on average by not exploring the novel option as often. This was advantageous because the novel options were replaced more often than expected, and thus had short time horizons, and therefore could not be exploited in the future. When environments are unstable, or time horizons are short, exploration does not pay off, because the options are not available in the future, and an impulsive strategy that prioritizes immediate rewards is more beneficial. Direct manipulation of the time horizon of available choices has shown a similar result and has shown that human subjects can adapt to the time horizon for options during an explore-exploit task [21]. However, past work investigating novelty-seeking in clinical groups has shown mixed outcomes. Clinical groups that rank high on impulsivity on self-report questionnaires have been shown to exhibit risk-seeking and novelty-seeking behaviors, but not in all cases [87], and in some patient populations, novelty-seeking and impulsivity are largely separable behaviors [88,89]. Past work with the Explore-Exploit task as we have simulated it here, has shown that as the discount factor increases in this model, the novelty bonus increases [44,85]. This novelty bonus can account for high rates of choosing the novel option among other options [84,85]. While the results here show less novelty-seeking for impulsive agents, the framework would allow for experiments that decouple these two features of decision-making. For example, we would predict that some clinical groups labeled as impulsive would perform similar to our computational impulsive agents and perform better than healthy controls in high substitution rate environments, while others would choose the novel options more often, which might hurt overall performance. By manipulating the task parameters, it would be possible to shed light on the interactions between impulsivity in clinical populations and novelty-seeking, which we have defined as exploration of options with unknown reward rates.

In all three tasks presented, we modeled impulsive choice behavior in the context of misestimation of the task environment and manipulated the discount factor that weights the value of future rewards. However, an individual in a laboratory task might exhibit preference for a smaller, more certain option either because it will come sooner (time preference) or because it is certain (risk preference). Past work has shown that individual attitudes toward risk might play an independent role from time preference in estimating the discount factor [90,91]. While we do not dissociate these two factors in our models, past work has incorporated preferences for time and risk into the discount factor term to improve estimates of discounting in human subjects [92].

Furthermore, it remains an open question whether individual preferences for immediate rewards are due to attitudes towards risk or due to an inability to learn transition probabilities to future rewards. While beyond the scope of this study, it is worth acknowledging the possibility that impulsive choices could arise from poor planning ability, or from a conscious devaluing of future expected values. However, recent work suggests that deficits in planning or goal pursuit might be separable from impulsive choice behavior, as human subjects labeled as impulsive can also exhibit goal-oriented behaviors that require extensive planning [93].

In summary, previous work suggests that impulsive decision-making in clinical groups is maladaptive [94,95]. In contrast, our results across the three tasks suggest that impulsive behavior is not inherently negative and can be beneficial when an environment is more volatile than expected. Therefore, impulsive choice patterns can be adaptively optimal. It is not the agent that is suboptimal, but the match between the environment to which an agent is adapted, and the environment in which an agent is being tested. Furthermore, the framework here makes predictions about how human subjects, labeled impulsive by self-report or other means, might perform better in a variety of decision-making tasks. While past work has suggested that delay and risk are not necessarily equitable or represented as a single construct at the neural level [37,96], past literature has operationalized impulsivity through discounting of future rewards and the discount factor [55,56,97]. By combining these three tasks into a single framework, united by the discount factor, it becomes possible to validate the consistency of the discount factor for human participants. We have demonstrated parameter regimes where impulsive agents could fare better than non-impulsive agents that could be used to test human participants. For example, if “impulsive” human participants exhibit impulsive choice in TD and Beads, but choose novel options much more than non-impulsive agents in the Explore-Exploit task, this would suggest that the discount factor should be reconsidered as a way to operationalize impulsive choice in the context of novelty.

There is a growing literature on how experience in resource-poor environments and early-life stress can lead to changes in decision-making behavior and to favoring immediate over future rewards [98–103], which suggests impulsive choice behavior might be an adaptation to environmental instability. Furthermore, accurate assessment of environmental controllability has been shown to improve with development and age, suggesting that some impulsive choice behavior might arise from a dysfunction during development [104]. Although impulsivity is often assumed to be a trait, it may be a state, perhaps slowly changing, and impulsive choice behavior might reflect the environment to which an agent has adapted. Future work should investigate the flexibility of patients to adapt to impulsive task environments. The computational framework presented here opens a variety of possibilities to understand impulsive choice behavior as a gradient, rather than a binary label, and to better understand how human subjects weigh immediate and future rewards in the contexts of monetary discounting, information sampling, and novelty seeking. We believe this framework allows for quantification of impulsive choice behavior in a new light that will be useful to clinicians and researchers investigating factors that lead to impulsive choices.

## Methods

All simulations and analyses described below were conducted using MATLAB.

### General algorithm

We first discuss aspects of the algorithm that are consistent across all tasks. Similar methods were used to analyze patient data in these same three tasks [43]. In the present manuscript we are carrying out theoretical analyses to simulate behavior preferences of different agents. Simulations of two of the tasks (Information sampling & 3-armed Bandit) were previously described [44]. We first summarize the basic framework, which is described in more detail in the two previous studies. We then describe the specifics of each task and the manipulations of the agent and the environment used to achieve varied levels of uncertainty to answer the question posed in this study.

All tasks involved considering immediate rewards and future rewards at each step without consideration of previous steps. Thus, all tasks can be modeled as Markov Decision Processes (MDP) or Partially Observed MDPs (POMDP). The MDP framework models the utility, *u*, of a state, *s*, at time *t* as

$$u_{t}(s_{t})={max}_{a\in A_{s_{t}}}\{Q(s_{t},a)\}$$ (1)

where $A_{s_{t}}$ is the set of available actions in state *s* at time *t*, *a* is an action, and *Q*(*s*_*t*_, *a*) is the action value. The action value is the combination of immediate reward, possible cost, and discounted expected future rewards:

$$Q(s_{t},a)=r(s_{t},a)+C(s_{t},a)+\gamma \sum_{j\in S}p(j|s_{t},a)u_{t+1}(j)$$ (2)

where *r*(*s*_*t*_, *a*) is the immediate reward received in state *s* at time *t* if action *a* is taken and *C*(*s*_*t*_, *a*) is the cost to sample. These quantities make up the immediate expected value (IEV), which is the reward (cost) that will be received in the current time step when an action is taken. The future expected value (FEV) is the discounted expected future rewards, given an action. The expectation is taken over all possible future states, *S*, at time *t + 1*. Each transition probability, *p*(*j*|*s*_*t*_, *a*), is the probability of transitioning to a particular state, *j*, from the current state if one takes action *a*. The discount factor, g, defines the discounting of future rewards and takes on values between 0 and 1. Thus the utility equation is a maximization across all possible actions to find the most valuable action to take.

For discrete state, finite horizon models with tractable state spaces (e.g. Temporal discounting & Information sampling), utility estimates can be calculated by backward induction [44,53,105]. Because there is a termination of the sequence of choices in these tasks and a defined final reward (outcome), we can start by defining the utilities at the final state(s). We can then work backward to define the utilities of the previous states. If *N* is the final state:

1. Set *t* = N

$$u_{N}(s_{N})=r(s_{N})\;for\;all\;s_{N}\in N$$ (3)

2. Substitute *t-1* and compute the utility:

$$u_{t}(s_{t})={max}_{a\in A_{s_{t}}}\{r(s_{t},a)+C(s_{t},a)+\gamma \sum_{j\in S}p(j|s_{t},a)u_{t+1}(j)\}$$ (4)

Then set:

$$A_{s_{t,t}}^{*}={argmax}_{a\in A_{s_{t}}}\{r(s_{t},a)+C(s_{t},a)+\gamma \sum_{j\in S}p(j|s_{t},a)u_{t+1}(j)\}$$ (5)

3. If *t* = 1, stop, otherwise return to 2.

The set $A_{s_{t,t}}^{*}$ contains all actions, *a*, which maximize the utility.

The Explore-Exploit task was modeled as an infinite horizon POMDP. Utilities were fit using the value iteration algorithm [44,53]. The algorithm starts by initializing a vector of utilities across states,*u*^0^, to random values, and then computing:

$${u(s_{t})}^{n+1}={max}_{a\in A_{s_{t}}}\{r(s_{t},a)+\gamma \sum_{j\in S}p(j|s_{t},a)u^{n}(j)\}$$ (6)

Because the state-space of the task was intractable over useful horizons, we used a B-spline basis function approximation [44] to estimate the utilities:

$$\hat{u}(s)=\sum_{i=1}^{M}b_{i}\phi_{i}(s)$$ (7)

where $\hat{u}(s)$ is the approximation of the utility, *b*_*i*_ are the basis coefficients, and *ϕ*_*i*_(*s*) are the basis functions. We then calculated a projection matrix, *H*, and the approximation:

$$\hat{u}=Hu$$ (8)

The approximation was plugged into the righthand side of Eq (6) in place of *u*^*n*^(*j*). Approximations to the new values were iteratively calculated until convergence:

$${\hat{u}}^{n+1}=Hu^{n+1}$$ (9)

### Manipulation of uncertainty

Agents built on MDPs optimize expected reward when they are matched to the statistics of the environment, where matched means that the parameters of the probability model on which the agent is built are the parameters of the environment from which the agent samples in the simulations [53]. Therefore, an impulsive MDP agent will outperform a non-impulsive agent when the non-impulsive agent is not as well matched to the statistics of the environment. Here we were interested in the trade-off between immediate and future expected value, as this is the trade-off assessed with experimental measures of impulsivity. Impulsive subjects overweight IEVs, relative to FEVs, because they prefer immediate to delayed rewards. Therefore, we considered mismatches between agents and environments in FEVs, which are products of the uncertainty of state transitions, *p*(*j*|*s*_*t*_, *a*), and discount factor, *γ*.

One way to approach this would be to show that when transition probabilities in the environment are more uncertain, i.e., when *p*(*j*|*s*_*t*_, *a*) in the environment is high entropy, agents that assume *p*(*j*|*s*_*t*_, *a*) is low entropy will do worse than agents that have the proper environmental model. However, this would not show differences in the discount factor as this would be true with matched discount factors. Behavioral measures of impulsivity used in the laboratory and descriptive definitions of impulsivity often use discount factors to characterize impulsive choices. Therefore, we chose an approach that would show that having a shorter time horizon, characterized by a smaller discount factor, can be beneficial when environment and agent expectations are not matched. Specifically, when environments are more uncertain than expected, impulsive choice strategies can be beneficial. After the description of each decision-making task and model, we describe how we modified the parameters that described the agent’s expectation and the parameters that described the environment in which the agent made choices to achieve a mismatch between the agent’s expectations and the actual environment. Thus, we modeled MDP agents using assumed uncertainty values, and subsequently used these agents to make choices in environments that had mismatched uncertainty values. We use subscripts of “agent” for MDP model parameters, and subscripts of “env” (to indicate environment), to refer to the statistics used to generate the actual outcomes on each trial. Thus, agents were not matched to their environments, and we examined the effect of this mismatch, and different discount factors, on the number of rewards received.

### Manipulation of impulsivity

Across all tasks, we use the discount factor, *γ*, to model impulsive and non-impulsive choice strategies. Impulsive agents are characterized by a low discount factor *γ*_*Impulsive* (*I*)_<0.7. Non-impulsive agents have a high discount factor *γ*_*Non−impulsive* (*NI*)_ = 0.99.

### Statistical analyses

To compare mean reward and choice behavior of pairs of agents, paired t-tests and paired sample t-tests were used as noted in the results. For the Explore-Exploit task, a two-way ANOVA was used to determine main effects of discount factor and substitution rate and interaction effects on mean reward and choice behavior. To compute effect sizes, we used Cohen’s d. When agents were given identical trials, we used Cohen’s d effect size for paired samples x1 and x2:

$$d=(\mu_{1}-\mu_{2})/\sqrt{var(\mu_{1}-\mu_{2})}$$ (10)

and when agents were given different trials, we used

$$d=(\mu_{1}-\mu_{2})/\sqrt{(s_{1}^{2}-s_{2}^{2})/2}$$ (11)

where *μ*_1_ and *μ*_2_ were the mean values and *s*_1_ and *s*_2_ were the sample standard deviations for reward or choice behavior for each agent. To provide guidance for using these tasks with human participants, we calculated the number of iterations (i.e. sample size) required to ensure that a comparison has a specified power, given the effect size observed. we used a power of β = 0.80, significance level α = 0.05 [106].

### Temporal Discounting task

In the Temporal Discounting task, an agent is given a choice between a smaller, immediate reward (*R*_1_) and a larger, delayed (and possibly probabilistic) reward (*R*_2_). The task comes in several variants. For example, the Kirby delayed discounting questionnaire includes questions like, “Would you prefer $54 today, or $55 in 117 days?” and “Would you prefer $55 today or $75 in 61 days?”[38]. Replies to these questions are used in decision making models to estimate discount factors. Extensive work has shown that reward value decreases with delay to reward [38,107–109]. Furthermore, even when an experiment suggests that delayed rewards will be certain, human participants select options with lower expected values more often when outcomes are immediate rather than delayed. When both options are offered with a delay, participants choose the option with the larger expected value, even if that delay is larger. Experiments combining manipulations of uncertainty (through probabilistic reward offers) and time delays show that manipulating uncertainty directly has little effect on the preferences for delayed rewards. These experiments suggest that human participants attribute uncertainty to delayed rewards [37].

To model this task, we used a previously published, quasi-hyperbolic discounting model [43,44,109]. We assume a state space in which an action *a* (choose immediate reward or choose delayed reward) leads to the immediate reward state (*s*_*IR*_) or a sequence of transition states (*s*_*b*_). Each transition state leads to the subsequent transition state, an intermediate terminal state (*s*_*a*_) that terminates the episode and results in no reward, or if it is the final transition state, the final reward state (*s*_*DR*_) in which *R*_2_ is received. The sequence of unrewarded states models the temporal delay to the second option and the uncertainty around one’s ability to reach the terminal delayed reward state (*s*_*DR*_). The transition probabilities are defined by two parameters: β, which parameterizes the transition probability of the first step at t = 0, and δ, which is the discretized transition probability between the sequential *s*_*b*_ transition states. Thus, the model implements the progression through the state space with the following probabilities:

The probability of moving to the next intermediate transition state at the start is:

$$p(s_{1}=s_{b})=\beta \delta$$ (12)

The probability of terminating in an exit state at the start is:

$$p(s_{1}=s_{a})=1-\beta \delta$$ (13)

The probability of moving to the next intermediate transition state given that we are in an intermediate transition state:

$$p(s_{t+1}=s_{b}|s_{t}=s_{b})=\delta$$ (14)

and the probability of terminating at an exit state, given that one is in an intermediate transition state is:

$$p(s_{t+1}=s_{a}|s_{t}=s_{b})=1-\delta$$ (15)

The value of the immediate reward is *R*_1_ and the value of delayed reward is Q(*a* = choose *R*_2_ at delay N) = *R*_2_*βδ*^*N*^. For the modeling in this study, *β* = 1 for all conditions, which makes the quasi-hyperbolic model equivalent to an exponential model. While MDPs inherently discount future rewards exponentially, past work has suggested that human behavior can be fit better by hyperbolic discounting [110–112]), and a value of *β*<1 would likely be more appropriate for fitting human behavioral data but would not affect the interpretation of the results presented here.

### Manipulation of uncertainty in the Temporal Discounting task

For the Temporal Discounting task, the transition probability, *δ*, was used to manipulate uncertainty. If *δ* = 0.5, exiting at an intermediate, unrewarded state is as likely as moving one step closer to the final reward state, and if *δ* = 0.9, progression to the next state happens 90% of the time. Uncertainty in the environment was modeled as a smaller value of *δ* for the expected transition probability to the delayed reward than the one used to calculate the state-action value for choosing the delayed reward in the agent’s model. The state-action value, *Q*(*s*_*t*_, *a*), was computed using δ_*agent*_ and the true outcomes were simulated using δ_*env*_, where δ_*agent*_<δ_*env*_ (certain environment) and δ_*agent*_>δ_*env*_ (uncertain environment). Thus, each δ, δ_*agent*_ and δ_*env*_, had two possible values of 0.55 and 0.99, although results are not contingent on these exact values. We compared the performance of two agents, one with a low discount factor (*γ*_*Impulsive*_ = 0.6) and one with a high discount factor (*γ*_*Non−Impulsive*_ = 0.99), to model impulsive and non-impulsive behavior, respectively.

To simulate outcomes across multiple trials, trials were generated using a range of unitless small reward sizes (*R*_1_ = 1: 0.5: 51) and large reward sizes (*R*_2_ = 50: 10: 1050), and unitless time delays (N = 1:20). For each trial, the action value *Q*(*s*_*t*_, *a*) was computed for the two options using the discount factor, *γ*_*agent*_, and δ_*agent*_ such that *Q*(*Choose R*_1_) = *R*_1_, and *Q*(*Choose R*_2_) = *R*_2_ *γ*_*agent*_^*N*^δ_*agent*_^*N*^. The agent then picked the larger action value which determined whether they received *R*_1_ or proceeded through the simulation of transition states towards *R*_2_ on that trial. To simulate transition states to the delayed reward, the series of probabilistic states were simulated using δ_*env*_ and N, such that the agent effectively proceeded through N Bernoulli trials with p = δ_*env*_ to determine whether *R*_2_ was received on that trial when *R*_2_ was selected, or no reward was received. Average reward was calculated across 10 iterations of 100 trials for each agent in each environment. We then compared the average reward received and frequency of choosing the larger, delayed option when δ_*agent*_<δ_*env*_ and when δ_*agent*_>δ_*env*_ for both agents.

### Information Sampling (Beads) task

In the information sampling task, participants are asked to guess the majority color of beads in an urn (one of two colors, for example, blue and green). Evidence for the majority bead color is accumulated one bead at a time, with a small cost for each bead drawn. At each time step, there are three possible actions: (a) guess green (b) guess blue or (c) draw another bead to gather more information. The state, s_t_, is given by the number of draws (*n*_*d*_) and the number of accumulated blue beads s_t_ = {n_d_, n_b_}. Each bead draw incurs a cost, *C*_*draw*_(*s*_*t*_,*a*), and there is a maximum number of allowable draws. This allowed us to model the task using a finite-horizon, finite state, POMDP [45]. Additional parameters include the true fraction of beads in the majority urn (q), the reward for guessing correctly (R_correct_) and the cost for guessing incorrectly (R_error_).

For a given trial, a bead draw sequence (of length max draws) was generated using the fraction of majority beads *q*. State-action values were calculated for each possible action for each step to determine when the agent should stop drawing and guess a majority color.

For guessing that the urn is majority blue:

$$r(s_{t},a=guess\;blue)=R_{error}p_{g}+R_{correct}p_{b}$$ (16)

where *p*_*b*_ is the probability the urn is majority blue, given by:

$$p_{b}={\left[1+{\left(\frac{q}{1-q}\right)}^{\left(n_{d}-2n_{b}\right)}\right]}^{-1}$$ (17)

and *p*_*g*_ is the probability the urn is majority green, given by *p*_*g*_ = 1−*p*_*b*_._._ For guessing an urn color, the second term in the MDP utility equation that represents the FEV is 0, as choosing an urn terminates the sequence of actions.

For drawing again, *a* = draw, we have:

$$Q_{t}(s_{t},a=draw)=C_{draw}+\gamma \sum_{j\in S}p(j|s_{t},a)u_{t+1}(j)$$ (18)

From a given state, s_t_, if the agent draws again, the two possible next states are s_t+1_ = {n_d_+1, n_b_+1} if a blue bead is drawn, or s_t+1_ = {n_d_ +1, n_b_} if a green bead is drawn. The corresponding transition probabilities are:

$$p(n_{d+1}+1,n_{b}+1|s_{t}=[n_{d},n_{b}],a=draw)=qp_{b}+(1-q)p_{g}$$ (19)

and

$$p(n_{d+1}+1,n_{b}|s_{t}=[n_{d},n_{b}],a=draw)=(1-q)p_{b}+{qp}_{g}$$ (20)

The action taken on each step was the one with the highest value. When the action value for guessing blue or green was higher than the action value for draw, the corresponding urn was chosen and total reward (whether the guess was correct or incorrect, and how many draws were taken) was computed. To model average agent behavior, 100 batches of 100 draw sequences were generated for each set of task parameters. Action values were computed for each step of each bead draw sequence and the agent picked the action associated with the largest action value at each step. Once the agent picked a color or reached the maximum number of draws (20 in these simulations), the reward collected and draw cost incurred was calculated and the number of draws before choice was recorded. This was conducted across all simulated sequences in a batch and average reward and average number of draws was calculated across the batches of bead draws. This was repeated for each discount factor across all task parameter sets.

### Manipulation of uncertainty in the Information Sampling (Beads) task

To vary the level of uncertainty in the beads task, three parameters were modified to create parametric environments where either a non-impulsive agent (higher discount factor, *γ*_*NI*_ = 0.99) or impulsive agent (lower discount factor, *γ*_*I*_ = 0.55) would obtain more overall reward. First, the fraction of majority beads, *q*_*env*_, used to generate the bead draw sequences was either higher or lower than the majority fraction used to calculate the agent’s state-action values, *q*_*agent*_. For example, if *q*_*env*_, used to generate the bead draws, was lower than *q*_*agent*_, then the agent would expect more information from each bead draw was present in the actual sequences. The second parameter modified was the cost to draw a bead (*C*_*draw*_). Varying *C*_*draw*_ affected whether the impulsive or non-impulsive agent collected more reward on average. Third, the cost of guessing incorrectly (*R*_*error*_) was set larger than the reward for guessing correctly (*R*_*correct*_). While there exists a parameter range where |*R*_*error*_| = |*R*_*correct*_| and the impulsive agent can collect more average reward, in this domain the agent typically only makes one draw before the action value for guessing one of the colors becomes greater than the action value for drawing a bead. If |*R*_*error*_|>|*R*_*correct*_|, then this encourages multiple draws from the impulsive agent, leading to a richer behavioral output.

### Explore-Exploit task

The Explore-Exploit task is a 3-armed bandit task in which one option is replaced with a novel option at a parametrized, stochastic rate. The size of the reward is the same for each option, but the probability of receiving a reward from each option differs. The agent must learn the value of each option through experience. After the agent experiences the three available options for a period, one of the options is randomly selected and replaced by a novel option. The agent must then decide whether to choose the novel option (explore) or select (exploit) one of the remaining two options with which the agent has more experience. The replacements are not known in advance and happen stochastically, so there is no way to plan for an option being replaced.

In the model states are defined by the number of times each option has been chosen and the number of times it has been rewarded *s*_*t*_ *=* {*R*_*1*_,*C*_*1*_, *R*_*2*_,*C*_*2*_, *R*_*3*_, *C*_*3*_}. The immediate reward estimate is given by:

$$r(s_{t},a=choose\;option\;i)=\frac{R_{i}+1}{C_{i}+2}$$ (21)

The numerator and denominator include the assumption of a beta(1,1) prior, reflecting an a-priori reward probability of 0.5. The set of possible next states is given by the chosen target, whether it was rewarded, and whether one of the options was replaced with a novel option. The probability of a novel substitution, *h* was a parameter and *q*_*i*_ = *r*(*s*_*t*_, *a* = *i*). The transition probability to a state without a novel choice substitution and no reward is given by:

$$p(\ldots ,C_{i}+1,R_{i},\ldots |s_{t}=[\ldots ,C_{i},R_{i},\ldots ],a=choose\;option\;i)=(1-q_{i})(1-h)$$

and if the chosen target was rewarded and there was still no novel option:

$$p(\ldots ,C_{i}+1,R_{i}+1,\ldots |s_{t}=[\ldots ,C_{i},R_{i},\ldots ],a=choose\;option\;i)=q_{i}(1-h)$$

When a novel option was introduced, it could replace the chosen option or a different option. If the chosen target, *i*, was not rewarded and a different target, *j*, was replaced, the transition probability is:

$$p(\ldots ,C_{i}+1,R_{i},C_{j}=0,R_{j}=0|s_{t}=[\ldots ,C_{i},R_{i},\ldots ],a=choose\;option\;i)=(1-q_{i})h/3$$

As long as the chosen target was not rewarded, the transition probability is the same, even if the chosen target, *i*, was replaced instead. Correspondingly, if the chosen target, *i*, was rewarded, and a different target, *j*, was replaced, the transition probability is given by:

$$p2(\ldots ,C_{i}+1,R_{i}+1,C_{j}=0,R_{j}=0|s_{t}=[\ldots ,C_{i},R_{i},\ldots ],a=choose\;option\;i)=q_{i}h/3$$

and is the same following reward and replacement of the chosen target, *i*.

### Manipulation of uncertainty in the Explore-Exploit task

To manipulate uncertainty in the Explore-Exploit task, we varied the substitution rate of the novel option. Similar to the mismatch method in the Information Sampling task, the agents had a single substitution rate (*p*_*agent*_ = 0.08) and the novel option substitution rate in the environment was varied from *p*_*env*_ = 0.02 to *p*_*env*_ = 0.2. Thus, the agents expected a substitution rate of 0.08, but in each experimental condition, the substitution rate in the environment was either higher than, lower than, or equal to the expected substitution rate. The low substitution rate represents a certain environment, where the values of the three options are stable for long periods. The high substitution rate represents an uncertain environment because there are frequent introductions of novel options and therefore any single option cannot be exploited for long periods.

To compare average reward collected for impulsive and non-impulsive agents, we varied the discount factor (*γ*) used to compute the action values for each of the three options. We simulated 50 iterations of 250,000 trials of three options. The underlying reward rates could be 0.8, 0.5 or 0.3, and when novel options were introduced their reward rate was assigned randomly. The agent had to explore novel options to learn their reward rates. Sets of available options could include any combination of these three reward probabilities. The novel options replaced one of the options at rate *p*_*env*_. We used the model to generate the action values for these trials. Choices were generated by selecting the largest action value for each trial. Rewards were calculated based on choosing these options and their underlying reward rates. To compare agents with different discount factors, identical sequences of trials were given to the two agents for each substitution rate.

To compare the balance between exploitation and exploration of the novel option, we calculated how often different agents selected the novel option on first appearance. This was calculated using the same choice data used to calculate average reward.

## Supporting information

S1 Fig Heatmaps of differences in average reward for non-impulsive and impulsive agents across a range of expected and actual transition probabilities in the Temporal Discounting task. Each panel is a heatmap showing the differences in average reward for a pair of non-impulsive and impulsive agents for a range of transition probabilities. δ_*agent*_ (x-axis) is the transition probability fed to the model and δ_*env*_ (y-axis) is the actual transition probability used to calculate the future expected values of the delayed rewards.
(PDF)

S2 Fig Model behavior across a range of Beads task parameters with even outcomes for correct and incorrect guesses (R_correct_ = 10, R_incorrect_ = -10). Each panel is a heatmap showing the differences in average reward for a pair of non-impulsive and impulsive agents, indicated by the discount factors on the far left. Each column has a set of heatmaps for the models’ expected majority fraction of beads, q_agent_. Each row has a set of heatmaps for a pair of discount factors (impulsive & non-impulsive). The x-axis of each heatmap is the draw cost and the y-axis is the difference between the model input q_agent_ and the majority fraction used to generate the bead draws, q_env_. More blue values indicate the non-impulsive agent collected more average reward and more red values indicate the impulsive agent collected more reward. As q_agent_ increases (left to right), the domain in which the non-impulsive agent performs better expands.
(PDF)
