# Supplementary material for: Environmental uncertainty and the advantage of impulsive choice strategies
Source: PLoS Comput Biol. 2023 Jan 30;19(1):e1010873. doi: 10.1371/journal.pcbi.1010873 (PMC9910799; doi:10.1371/journal.pcbi.1010873)
Supplement: S1 Fig — Each panel is a heatmap showing the differences in average reward for a pair of non-impulsive and impulsive agents for a range of transition probabilities. δagent (x-axis) is the transition probability fed to the model and δenv (y-axis) is the actual transition probability used to calculate the future expected values of the delayed rewards. (PDF) [file pcbi.1010873.s001.pdf]

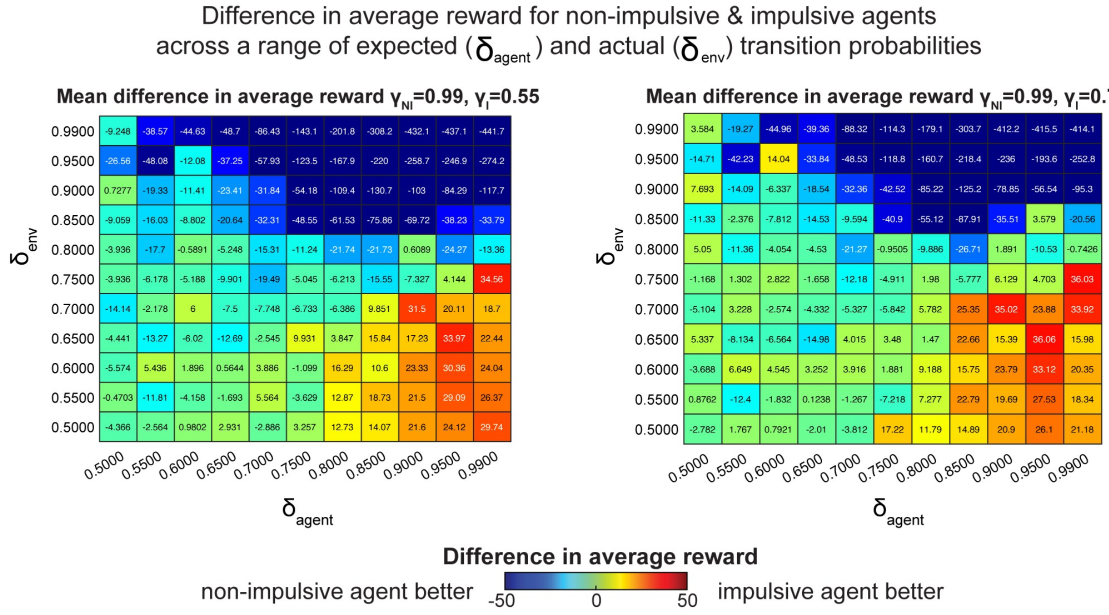

S1 Fig. Heatmaps of differences in average reward for non-impulsive and impulsive agents across a range of expected and actual transition probabilities in the Temporal Discounting task. Each panel is a heatmap showing the differences in average reward for a pair of non-impulsive and impulsive agents for a range of transition probabilities. δ_{agent} (x-axis) is the transition probability fed to the model and δ_{env} (y-axis) is the actual transition probability used to calculate the future expected values of the delayed rewards.
