# Supplementary material for: Environmental uncertainty and the advantage of impulsive choice strategies
Source: PLoS Comput Biol. 2023 Jan 30;19(1):e1010873. doi: 10.1371/journal.pcbi.1010873 (PMC9910799; doi:10.1371/journal.pcbi.1010873)
Supplement: S2 Fig — Each panel is a heatmap showing the differences in average reward for a pair of non-impulsive and impulsive agents, indicated by the discount factors on the far left. Each column has a set of heatmaps for the models’ expected majority fraction of beads, qagent. Each row has a set of heatmaps for a pair of discount factors (impulsive & non-impulsive). The x-axis of each heatmap is the draw cost and the y-axis is the difference between the model input qagent and the majority fraction used to generate the bead draws, qenv. More blue values indicate the non-impulsive agent collected more average reward and more red values indicate the impulsive agent collected more reward. As qagent increases (left to right), the domain in which the non-impulsive agent performs better expands. (PDF) [file pcbi.1010873.s002.pdf]

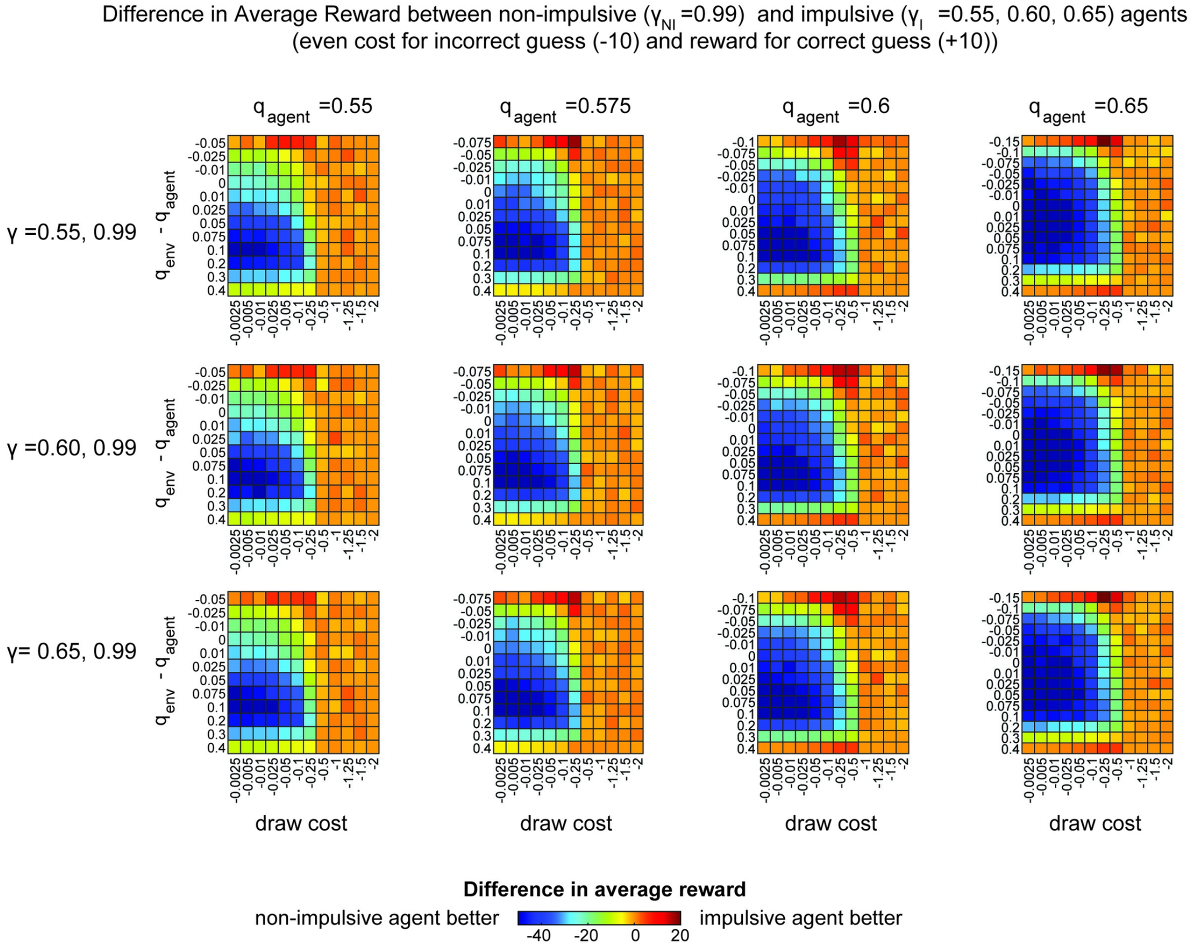

S2 Fig. Model behavior across a range of Beads task parameters with even outcomes for correct and incorrect guesses ($R_{correct}$= 10, $R_{incorrect}$= -10). Each panel is a heatmap showing the differences in average reward for a pair of non-impulsive and impulsive agents, indicated by the discount factors on the far left. Each column has a set of heatmaps for the models' expected majority fraction of beads, q_{agent}. Each row has a set of heatmaps for a pair of discount factors (impulsive & non-impulsive). The x-axis of each heatmap is the draw cost and the y-axis is the difference between the model input q_{agent} and the majority fraction used to generate the bead draws, q_{env}. More blue values indicate the non-impulsive agent collected more average reward and more red values indicate the impulsive agent collected more reward. As q_{agent} increases (left to right), the domain in which the non-impulsive agent performs better expands.
